# Development and Evaluation of Egg-Free Mayonnaise Stabilized with Aquafaba and Gum Tragacanth: Functional, Sensory, and Storage Properties

**DOI:** 10.3390/molecules30173511

**Published:** 2025-08-27

**Authors:** Bakhtawar Shafique, Mian Anjum Murtaza, Muhammad Salman Farid, Kashif Ameer, Muhammad Imran Hussain, Monika Sienkiewicz, Anna Lichota, Łukasz Łopusiewicz

**Affiliations:** 1 Institute of Food Science and Nutrition, University of Sargodha, Sargodha 40100, Pakistan; bakhtawarshafique111@gmail.com (B.S.); anjum.murtaza@uos.edu.pk (M.A.M.); kashif.ameer@uos.edu.pk (K.A.); 2 College of Food Science and Technology, Shanghai Ocean University, Shanghai 201306, China; 3 Institute of Pharmacy, Department Pharmaceutical Biology, Greifswald University, Friedrich-Ludwig-Jahn-Str. 17, 17489 Greifswald, Germany; salmanfarid9187@gmail.com; 4 Department of Human Nutrition and Dietetics, Rashid Latif Khan University, Lahore 54600, Pakistan; imran.hussain@rlku.edu.pk; 5 Department of Pharmaceutical Microbiology and Microbiological Diagnostics, Medical University of Lodz, 90-151 Łódź, Poland; monika.sienkiewicz@umed.lodz.pl; 6 School of Medical & Health Sciences, Vizja University, 59 Okopowa Str., 01-043 Warsaw, Poland

**Keywords:** chickpea, egg replacer, vegan, plant-based emulsion, stability analysis

## Abstract

This study developed and evaluated plant-based mayonnaise formulations in which egg yolk was replaced with aquafaba (15–25%) and stabilized with gum tragacanth (0.3–1.0%). Formulations were prepared using canola oil and stored at 4 °C for 28 days. Aquafaba extract was characterized for total phenolic content (TPC) and total flavonoid content (TFC), while mayonnaise samples were assessed for physicochemical composition, creaming index, antioxidant activity, viscosity, texture, sensory properties, and microbiological stability. Total phenolic content (TPC) rose from 17.52 mg GAE/g at 10 µg to 135.34 mg GAE/g at 100 µg (*p* < 0.05), while total flavonoid content (TFC) increased from 76.95 to 192.42 mg TE/g over the same concentration range. These increases demonstrate the high antioxidant potential of aquafaba extract. The 25% aquafaba + 1% gum tragacanth formulation (T_3_) showed the highest protein content, viscosity, firmness, and antioxidant capacity, with improved storage stability compared to the control. FTIR analysis identified functional groups such as phenols, esters, and carboxylic acids, suggesting contributions to antioxidant activity and emulsion stability. Sensory evaluation indicated strong acceptance for T_3_. These results demonstrate that aquafaba combined with gum tragacanth can effectively replace egg yolk while maintaining desirable quality attributes.

## 1. Introduction

Mayonnaise is an oil-in-water emulsion typically containing 70–80% oil, stabilized by egg yolk. Conventional formulations are high in cholesterol and saturated fats, and the use of raw eggs poses a *Salmonella* contamination risk. Egg allergy is one of the most prevalent food allergies in children, typically developing in infancy and early childhood. It is triggered by proteins such as ovalbumin and ovomucoid in egg white, which can cause reactions ranging from mild skin rashes to severe anaphylaxis [1,2,3,4]. These health and safety concerns have driven interest in plant-based alternatives that maintain desirable texture, flavor, and stability while reducing fat and eliminating animal-derived ingredients. The global rise in veganism and flexitarian diets has fueled interest in egg-free mayonnaise, as consumers increasingly seek sustainable, cholesterol-free, and allergen-friendly products. Aquafaba, the viscous liquid from cooking chickpeas, exhibits emulsifying, foaming, and thickening properties [5,6,7]. It contains 92–95% water and 5–8% dry matter, including low-molecular-weight proteins, saponins, and carbohydrates [7,8]. It has been successfully applied as an egg replacer in bakery, confectionery, and emulsion-based products [9]. Gum tragacanth, a polysaccharide exudate from *Astragalus* spp., is a non-toxic, biodegradable, and pH-stable thickener and emulsifier [10,11,12,13]. Its high viscosity and ability to stabilize emulsions make it a promising candidate for improving texture and storage stability in plant-based mayonnaise.

Recent studies have investigated other plant-based stabilizers, such as pea protein–xanthan gum conjugates [14], yellow mustard gum [15], and chia mucilage–gum arabic blends [16], demonstrating good stability and consumer acceptance. However, research on aquafaba–gum tragacanth combinations is limited. The Scientific Committee for Food of the European Community has approved gum tragacanth as an additive with the E413 E-number in Europe. GT has a moisture content of 8.79–12.94 g/100 g for different species and produces more viscous solutions when added to water [17]. The protein content of GT varies based on species; for example, *A. compactus*, *A. microcephalus*, and *A. fluccosus* may contain 1.65–2.59% protein. Moreover, the carbohydrate content may vary between 83.81% and 86.52% for different species. Furthermore, GT species have variations in the mineral content, such as potassium and calcium, which are the major inorganic elements for species. Tragacanthic acid and tragacanthin are the two soluble fractions of GT, and they dissolve and yield the production of viscous colloidal hydrosols when dispersed in water, whereas bassorin (60–70%) is developed as an insoluble gel fraction [18]. One of the notable advantages of GT is its remarkable stability over a wide range of temperatures and pH levels. This characteristic ensures that the texture and consistency of plant-based mayonnaise remain stable during processing and storage. To stabilize the emulsion, it reduces the collision and droplet attachment by decreasing the movement and release of dispersed droplets in the emulsion.

Considerable research has been devoted to developing low-fat mayonnaise using various ingredients, primarily to reduce the negative health impacts associated with high oil content. GT was added at lower concentrations to facilitate the production of healthier mayonnaise formulations. Pedramnia, Elhamirad and Habashi [1] prepared mayonnaise with whey protein concentrates as an egg substitute and gum tragacanth as a fat substitute. The textural properties of mayonnaise improved with increased firmness and consistency, resulting in better adhesiveness and smaller oil droplet diameters. With the addition of gum tragacanth, a complex and strong gel-like structure was formed in the continuous phase of mayonnaise. However, GT exhibits limited solubility in cold water and requires prolonged hydration times to achieve full functionality. Shen, et al. [19] studied the emulsifying properties of pea protein conjugated with guar gum (G-PPI) as a functional emulsifier in mayonnaise. Compared to unmodified pea protein, G-PPI emulsions exhibited higher stability (89.4%), smaller droplet size (934.4 nm), and greater viscosity (48.62 mPa·s) [19]. Odep, et al. [20] investigated the use of gum arabic (GA) combined with chia mucilage as egg and fat replacers in fat-reduced mayonnaise formulations. Mayonnaise samples with chia mucilage (15–60%) and 3% GA showed significantly lower calories, reduced fat, and increased moisture than the controls [20]. Hashemi, et al. [21] studied the conjugation of lysozyme (Lyz) with gum arabic (GA) to enhance its emulsifying and antibacterial properties in mayonnaise. The Lyz-GA conjugate exhibited improved antibacterial activity against *Staphylococcus aureus* and *Escherichia coli* and enhanced emulsification stability and rheological properties, including elasticity and shear-thinning behavior. Sensory evaluation confirmed that the mayonnaise with the conjugate matched commercial standards, demonstrating its potential as a natural preservative and emulsifier in food products [21]. Liu, et al. [22] explored yellow mustard gum (YMG) as an emulsifier in vegan mayonnaise (VM) formulations at 0–1.0% concentrations. YMG significantly improved emulsion stability, rheology, droplet size, texture, and shelf-life compared to commercial products, making it a promising candidate for plant-based and clean-label applications [22]. Recent work has explored aquafaba as an egg replacer in vegan mayonnaise due to its foaming and emulsifying properties. For example, a recent study demonstrated that chickpea aquafaba can produce mayonnaise with good rheological stability and acceptable sensory properties [23].

Replacing egg yolk with aquafaba and stabilizing the emulsion with gum tragacanth would yield a plant-based mayonnaise with physicochemical stability, antioxidant potential, and sensory acceptability comparable to or exceeding those of conventional egg-based mayonnaise. Most previous studies have focused either on aquafaba or on alternative gums in isolation, without systematically examining their combined functionality. The present study differs by incorporating both aquafaba and gum tragacanth (GT) in defined ratios to optimize emulsion stability and nutritional quality. In addition to proximate composition and storage stability, this study also evaluates antioxidant activity, rheological behavior, and sensory acceptability, providing a broader functional assessment than earlier work. Therefore, the aim of this research was to develop plant-based mayonnaise formulations using aquafaba as an egg replacer and gum tragacanth as a natural stabilizer, and to evaluate their physicochemical, antioxidant, rheological, microbiological, and sensory characteristics during refrigerated storage. By integrating both functional ingredients, this study seeks to provide new insights into sustainable, allergen-free, and health-oriented condiment formulations.

## 2. Results and Discussion

### 2.1. Total Phenolic Content (TPC) and Total Flavonoid Content (TFC) in Aquafaba Extracts

**Figure 1:**
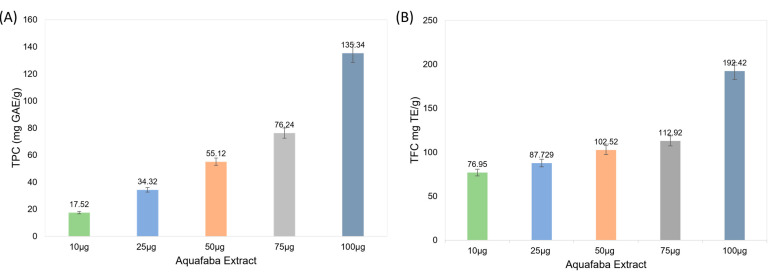
(**A**) Total phenolic content (TPC, mg GAE/g) and (**B**) total flavonoid content (TFC, mg TE/g) of aquafaba extract at different concentrations. Values represent mean ± SD (*n* = 3).

Phenolic compounds and flavonoids are key phytochemicals known for their antioxidant and therapeutic properties, and their quantification is essential for evaluating plant-based bioactivity [24]. Figure 1 shows the TPC (mg GAE/g) and TFC (mg TE/g) present in different concentrations of aquafaba extract. The TPC analysis revealed a concentration-dependent increase, with the highest value observed at 100 µg of extract (135.34 mg GAE/g) and the lowest value at 10 µg (17.52 mg GAE/g). This suggests that aquafaba extract contains a substantial number of phenolic compounds, particularly at higher concentrations. The extract showed the highest flavonoid content at 100 µg (192.42 mg TE/g), whereas the lowest value was found at 10 µg (76.95 mg TE/g). The results regarding the total flavonoid and total phenolic contents in the aquafaba extracts are presented in Tables S1 and S2. Two-way ANOVA results confirmed statistically significant differences (*p* < 0.05) in both TPC and TFC across concentrations, indicating dose-dependent enrichment of phenolic and flavonoid compounds. As shown in Figure 1, the total phenolic content (TPC) increased from 17.5 mg GAE/g at 10 µg concentration to 135.3 mg GAE/g at 100 µg, while the total flavonoid content (TFC) rose from 77.0 to 192.4 mg TE/g, confirming the strong antioxidant potential of aquafaba extract.

### 2.2. Anti-Oxidant Analysis

**Figure 2:**
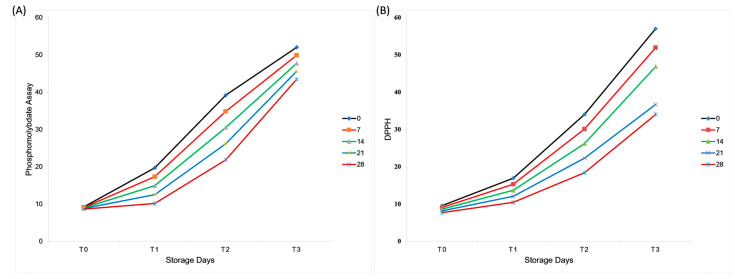
Effect of aquafaba and gum tragacanth on antioxidant activity of plant-based mayonnaise during 28 days of refrigerated storage, as determined by (**A**)phosphomolybdate assay and (**B**) DPPH radical scavenging assay. Values are expressed as mean ± SD (*n* = 3).

Phosphomolybdate and DPPH assays were conducted to evaluate the antioxidant activity of the mayonnaise samples over different storage periods (0, 7, 14, 21, and 28 d). Both assays showed highly significant differences (*p* < 0.05) among the treatments, storage durations, and their interactions. Treatment T_3_ consistently exhibited the highest antioxidant activity across both assays, with 52.02 ± 1.29 ^a^ (phosphomolybdate) and 57.01 ± 1.29 ^a^ (DPPH) on day 0. Antioxidant capacity declined over time, with the lowest values in treatment T_0_ on day 28 (8.65 ± 1.29M, phosphomolybdate; 7.69 ± 1.29O, DPPH). Plant extracts exerted a protective role in the oxidative stability of mayonnaise emulsions. The rancidity resistance was observed to be 33% and 58% with an increase in storage days. Incorporation of natural antioxidants improved the lipid oxidation resistance of fat emulsions. Sørensen, et al. [25] reported that incorporation of catechol with gum inhibited the oxidation rate. DPPH was used to determine the antioxidant capacity of the plant extracts [25]. Figure 2 shows that antioxidant activity, measured by phosphomolybdate and DPPH assays, was consistently higher in aquafaba–gum formulations, particularly T_3_, compared with the control, demonstrating the functional contribution of aquafaba and gum tragacanth.

### 2.3. Physicochemical Analysis of Plant-Based Mayonnaise

**Figure 3:**
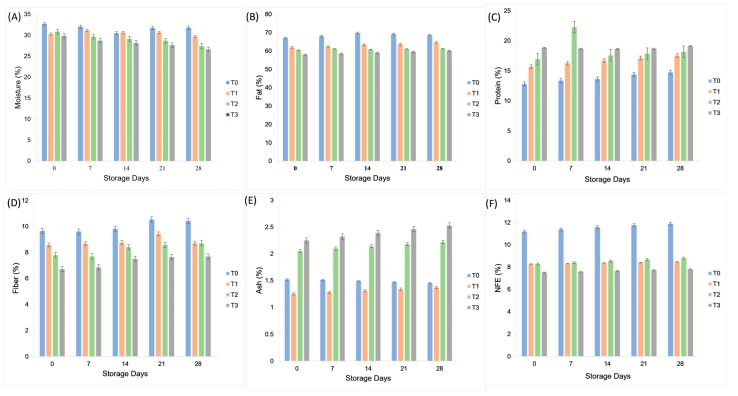
Effect of aquafaba and gum tragacanth on the proximate composition of control (T_0_) and plant-based mayonnaise (T_1_–T_3_) during 28 days of refrigerated storage. Parameters include (**A**) moisture (%); (**B**) fat (%); (**C**) protein (%); (**D**) fiber (%); (**E**) ash (%); and (**F**) carbohydrate (NFE, %). Values are expressed as mean ± SD (*n* = 3).

The moisture, fat, fiber, and nitrogen-free extract content of plant-based mayonnaise samples decreased as aquafaba and gum tragacanth concentrations increased, whereas protein and ash contents showed the opposite trend. Protein rose significantly from 12.82 ± 2.15% in the control (T_0_) to 19.13 ± 1.95% in T_3_ (*p* < 0.05), reflecting the contribution of aquafaba solids, which are rich in low-molecular-weight proteins and saponins. Similarly, ash content increased by 28% in T_3_ compared to T_0_, attributable to the mineral fraction of gum tragacanth, which contains potassium and calcium salts. The reduction in fat content across T_1_–T_3_ is explained by the partial substitution of the oil phase with aquafaba and gum solutions, which dilute lipid concentration and replace fat droplets with biopolymer matrices. Moisture decline during storage (0–28 day) is consistent with evaporation and water redistribution within the emulsion, leading to tighter microstructural binding of water by polysaccharides. These mechanisms align with previous findings where plant-based ingredients altered proximate composition by binding water and displacing oil. Evanuarini and Susilo [26] conducted a similar study in which flour of banana peel decreased the moisture content of plant-based mayonnaise. The control T_0_ treatment contained maximum moisture owing to the presence of 70% oil and no addition of banana flour. Banana peel flour has been shown to enhance the thickness of plant-based mayonnaise [26]. Mustafa, He, Shim and Reaney [5] reported similar results by determining the fiber, ash content and nitrogen-free extracts [5]. Ma and Boye [27] conducted a similar study in which protein and fat content was determined in an emulsified product. The results showed an increase in protein content and a decrease in fat content in plant-based mayonnaise due to the addition of chickpea protein [27]. Similarly, the protein content increased in T_1_, T_2_, and T_3_ due to the addition of aquafaba, a protein source from the pulse family. Fat content can be decreased by substituting fat droplets with numerous biopolymers, such as gums. In this study, the fat content decreased in T_1_, T_2_, and T_3_ due to the addition of canola and soybean oils. Figure 3 illustrates the effect of aquafaba and gum tragacanth on proximate composition during 28 days of storage. Compared to the control (T_0_), the T_3_ formulation retained higher protein and ash contents while showing reduced fat and moisture, reflecting the nutritional impact of aquafaba–gum enrichment.

**Figure 4:**
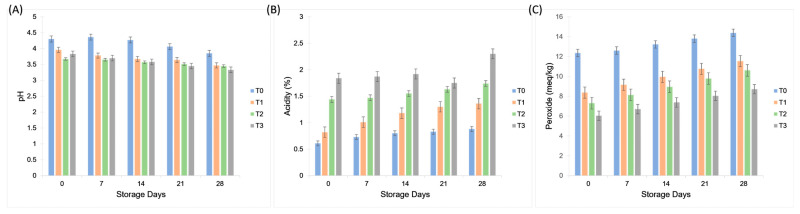
Effect of gum tragacanth and aquafaba on (**A**) pH; (**B**) acidity; and (**C**) peroxide value (meq/kg) of plant-based mayonnaise during 28 days of storage at 4 °C. Values represent mean ± SD (*n* = 3).

The initial pH values of the control sample (T_0_) and the other formulated treatments (T_1_, T_2_, and T_3_) were different (*p* < 0.05). The highest overall pH was recorded in the control sample (T_0_) at 0 d of storage (4.3 ± 0.13), whereas the lowest pH was noted for T_3_ (containing 25% aquafaba extract and 100% gum tragacanth) at 28 d of storage (3.33 ± 0.10). The decreasing trend in pH values over time indicates a gradual increase in acidity during refrigerated storage. Gum has a slightly acidic pH value. The acidity of the gum caused a reduction in the pH of the plant-based mayonnaise samples. With an increase in storage time, the acidity increases, which controls microbial growth. Therefore, gum can decrease microbial activity due to the increased acidity. In interaction, highest acidity was obtained for treatment T_3_ at 28th day of storage (2.3 ± 0.28 ^a^), whereas the lowest acidity was obtained for treatment T_0_ at 0 storage days (0.61 ± 0.12 ^l^). Mayonnaise had a high acidity value owing to the presence of gum at different concentrations. The high acidity was constant with pH value, which induced a reduction in the pH of the emulsion with an increase in the acidity. Ali and El Said [28] developed plant-based mayonnaise with the addition of gum arabic and results confirmed that GA is a complex polysaccharide, branched-chain, slightly acidic. Gum arabic was found to be a mixture of polysaccharides with potassium, magnesium, and calcium salts. They reported similar results, in which the samples showed an increase in acidity. The highest peroxide value was obtained for treatment T_0_ on the 28th day of storage (14.38 ± 0.84 ^a^), whereas the lowest peroxide value was obtained for treatment T_3_ on day 0 of storage (6.03 ± 0.84 ^l^). Orgulloso-Bautista, et al. [29] prepared plant-based mayonnaise with basil leaves which was less than 10 meq/kg at the end of storage day. The PV values for all mayonnaise samples exceeded the safety limit at the end of the storage period, while the plant-based mayonnaise sample reached only 6.88 meq/kg, indicating the prevention of oil oxidation. Egg was replaced with gum arabic, which resulted in the prevention of oil oxidation at 5 °C during the storage period. Figure 4 shows that pH gradually decreased, while acidity and peroxide values increased during storage. These changes were more pronounced in aquafaba–gum formulations than in the control, reflecting the impact of plant-based stabilizers on oxidative stability.

### 2.4. Stability in Plant-Based Mayonnaise

The highest creaming index was obtained for T_0_ at 28th day of storage (1.23 ± 0.03 ^a^), whereas the lowest creaming index was obtained for T_0_, T_1_, T_2_, and T_3_ at 0 d storage. The increase in creaming during storage reflects gravitational separation caused by droplet flocculation and water redistribution. In contrast, aquafaba–gum tragacanth formulations showed markedly lower creaming rates, particularly T_3_, due to the combined effects of polysaccharide-induced viscosity enhancement and protein–polysaccharide interfacial interactions. Gum tragacanth forms a three-dimensional hydrocolloid matrix that slows droplet mobility, while aquafaba proteins adsorb at the oil–water interface, forming a viscoelastic film that resists droplet coalescence. Together, these mechanisms reduce creaming by stabilizing dispersed droplets against gravitational separation. McClements and J. [30] reported that the incorporation of gum arabic into mayonnaise had a defending impact on emulsion separation into two layers compared to the control after 30 days at 5 °C storage temperature. The prompt increase in viscosity due to GA maintained the fluid and reduced the creaming rate.

**Figure 5:**
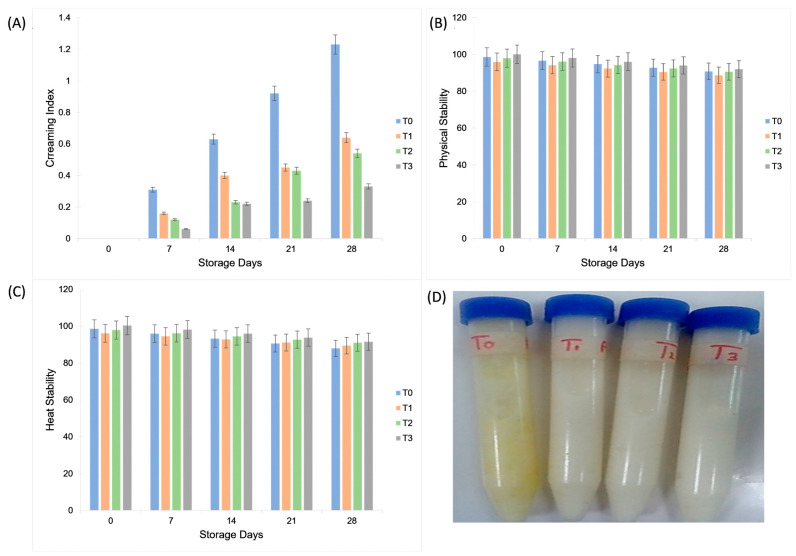
Effect of aquafaba and gum tragacanth on the (**A**) creaming index (%); (**B**) physical stability (%); and (**C**) heat stability (%) of (**D**) control and plant-based mayonnaise samples during 28 days of refrigerated storage. Values are expressed as mean ± SD (*n* = 3).

The maximum overall physical stability was observed for treatment T_3_ (96.08 ± 3.16 ^a^) among the various treatments, whereas during the storage period, the maximum overall physical stability was observed on day 0 of storage (98.2 ± 1.9 ^a^). Ali and El Said [28] reported that gum arabic exhibited great potential as an emulsifier till the end of storage. The plant-based mayonnaise exhibited maximum stability of 90.65%. Creaming stability in mayonnaise containing gum tragacanth (87.8–88.5%) remained comparable to the control (88.2%, *p* > 0.05), indicating that gum tragacanth effectively stabilized the emulsion to the same extent as egg yolk [28]. Similarly, the maximum overall heat stability was observed for treatment T_3_ (95.96 ± 3.38 ^a^) among various treatments, whereas during the storage period, the maximum overall heat stability was observed on day 0 of storage (98.22 ± 1.93 ^a^). As shown in Figure 5, aquafaba–gum tragacanth formulations exhibited higher creaming index and better heat stability compared with the control, confirming the role of gum in enhancing emulsion stability during storage.

### 2.5. Mold Count

**Figure 6:**
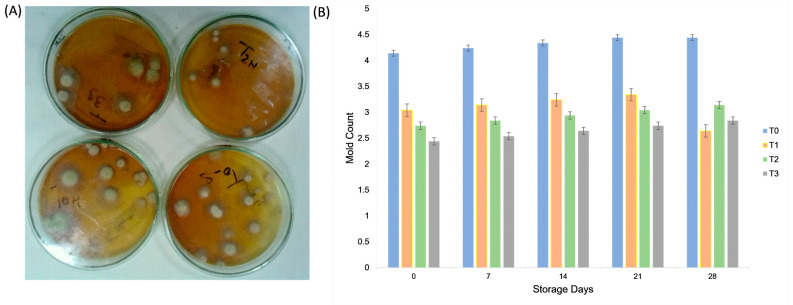
(**A**,**B**) Effect of aquafaba and gum tragacanth on mold counts (log CFU/g) of plant-based mayonnaise during 28 days of refrigerated storage. Values are expressed as mean ± SD (*n* = 3).

The mold count of the mayonnaise samples was evaluated over a 28 d storage period. The maximum overall mold count was observed for treatment T_0_ (4.32 ± 1.1 ^a^) among the various treatments, whereas during the storage period, the maximum overall mold count was observed on the 28th storage day (3.27 ± 1.32 ^a^). Amin, et al. [31] reduced the microbial count to 2%. At T_1_, no counts were detected with EE (0.5 and 258%) and AE (0.5%). The mold count increased at 20 °C storage, which was followed by hygienic–sanitary regulation, and the upper limit was established at 10 × 2 CFU/g. The mold count value increased regularly during storage in 25% of gum arabic samples. Mayonnaise samples exhibited no microbial growth in 50, 75, and 100% GA [31]. As shown in Figure 6, mold counts increased gradually during storage in all treatments; however, aquafaba–gum tragacanth formulations (especially T_3_) showed lower microbial growth compared with the control, indicating enhanced microbial stability.

### 2.6. Viscosity

The viscosity of the mayonnaise samples was evaluated on days 0, 7, 14, 21, and 28. The maximum overall viscosity was observed for treatment T_3_ among various treatments, whereas during the storage period, the maximum overall viscosity was observed on 0 storage day. The viscosity generally decreased over time, indicating a reduced thickness during storage. Viscosity rose from 8.44 ± 6.96 Pa·s in samples containing 0.3% gum tragacanth to 16.64 ± 2.57 Pa·s at 1.0% gum concentration (*p* < 0.05), showing that gum concentration enhanced emulsion thickness. However, viscosity declined steadily over storage, dropping to 9.3 Pa·s after 28 days, indicating gradual thinning of the emulsion due to droplet coalescence. Although gum tragacanth demonstrated good stabilizing capacity in plant-based mayonnaise, its limited solubility in cold water requires prolonged hydration (often several hours) to achieve full functional performance. This property, while acceptable in laboratory-scale trials, may present challenges for industrial application, where rapid processing is preferred. Future studies should therefore examine hydration optimization strategies, such as pre-soaking, controlled temperature treatments, or alternative hydrocolloids, to improve process efficiency without compromising product quality.

### 2.7. Textural Analysis

**Figure 7:**
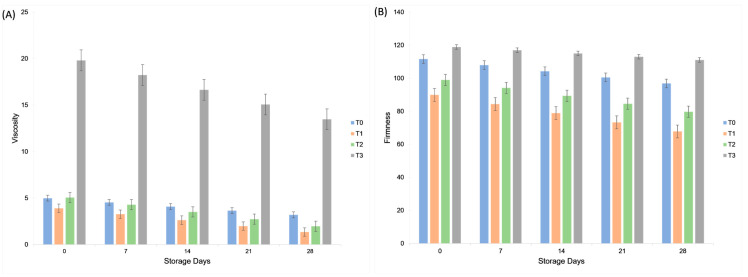
Effect of aquafaba and gum tragacanth on (**A**) viscosity (Pa·s) and (**B**) firmness (g) of plant-based mayonnaise during 28 days of refrigerated storage. Values are expressed as mean ± SD.

The firmness of the mayonnaise samples improved with the incorporation of gum tragacanth, which significantly influenced their textural properties. Treatment T_3_ showed the highest overall firmness (114.95 ± 3.08 ^a^), with a peak value on day 0 (118.88 ± 1.29 ^a^). The lowest firmness was recorded for T_0_ on day 28 (96.79 ± 1.29 ^h^). These findings align with Pedramnia, Elhamirad and Habashi [1], who reported the maximum and minimum adhesiveness, consistency, and firmness of mayonnaise samples, respectively. Regression measurements of textural firmness, adhesiveness, and cohesiveness showed that the examined samples suited the model well, with determination coefficients of 98.72, 99.96, and 99.78, respectively. The percentage of firmness improved when the egg yolk stabilizer was substituted with 0.3% gum tragacanth [1]. Figure 7 shows that viscosity and firmness were highest in the aquafaba–gum formulations, particularly T_3_, at day 0. Both parameters declined during storage, but plant-based samples maintained higher values than the control, indicating improved structural stability.

### 2.8. Fourier-Transform Infrared Spectroscopy (FTIR) Analysis of Mayonnaise Samples

**Figure 8:**
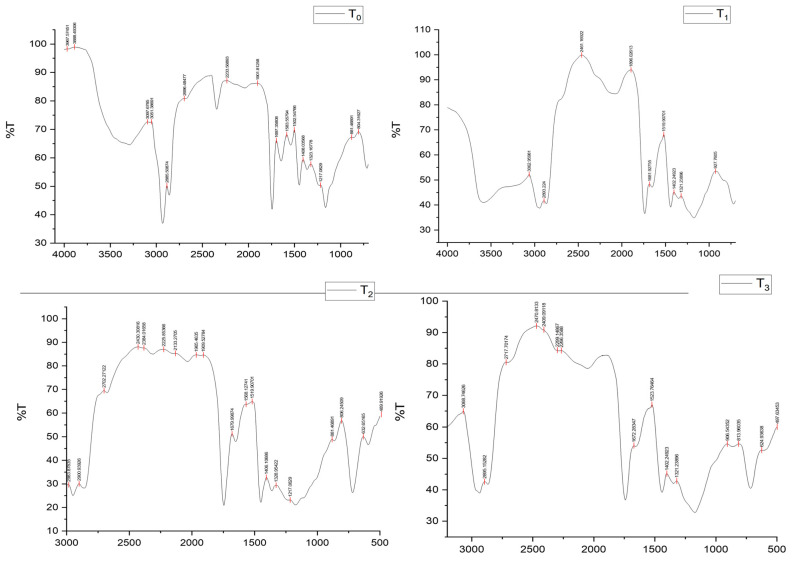
FTIR spectra of control mayonnaise (T_0_) and plant-based mayonnaise samples (T_1_, T_2_, T_3_). Distinct absorption peaks were observed in the regions corresponding to esters (1743 cm^−1^), phenols (3440 cm^−1^), and carboxylic acids (1630 cm^−1^), indicating the presence of functional compounds in aquafaba–gum formulations. Spectra represent average curves of triplicate measurements.

FTIR spectra of control mayonnaise (T_0_) and plant-based formulations (T_1_, T_2_, and T_3_) are presented. Across all samples, characteristic peaks were observed that correspond to major functional groups present in proteins, lipids, and polysaccharides. A strong absorption band at 3440 cm^−1^ was assigned to O–H stretching vibrations of phenols and hydroxyl groups, indicating the presence of antioxidant compounds. The band at 1743 cm^−1^ corresponded to ester C=O stretching, typically associated with lipids and emulsifying molecules, while a distinct absorption at 1630 cm^−1^ reflected carboxylic acid groups and amide I stretching in proteins. Additional peaks at 1429, 1516, and 1612 cm^−1^ were attributed to aromatic C=C, C=O, and C–C stretching, respectively, while bending vibrations of phenolic O–H groups were evident at 1359 cm^−1^. Differences among treatments were evident in peak intensities. The control sample (T_0_) exhibited lower absorbance at phenolic O–H (3440 cm^−1^) and carboxylic acid (1630 cm^−1^) regions compared to T_2_ and T_3_, reflecting the higher content of aquafaba-derived phenolic compounds in plant-based formulations. Similarly, ester-related bands (1743 cm^−1^) were more pronounced in T_3_, suggesting enhanced lipid–gum interactions contributing to emulsion stability. The spectra of T_1_ and T_2_ displayed intermediate values, consistent with their lower aquafaba and gum concentrations. These findings indicate that aquafaba and gum tragacanth contribute additional functional compounds such as phenolics, esters, and carboxylic acids that are absent or less prominent in egg-based mayonnaise. Their presence likely enhanced antioxidant activity and emulsion stability in plant-based samples, supporting the functional role of these ingredients in mayonnaise formulation. Thus, FTIR provides molecular-level confirmation that aquafaba–gum systems not only act as egg replacers but also enrich the product with bioactive components that improve quality and stability. The FTIR peak values in this study closely matched those reported by Khalid, et al. [32]. Figure 8 presents the FTIR spectra of the control (T_0_) and aquafaba–gum formulations (T_1_–T_3_). Characteristic peaks corresponding to esters, phenols, and carboxylic acids were observed, confirming the presence of functional groups associated with emulsifying and antioxidant properties.

### 2.9. Sensory Evaluation of Plant-Based Mayonnaise

**Figure 9:**
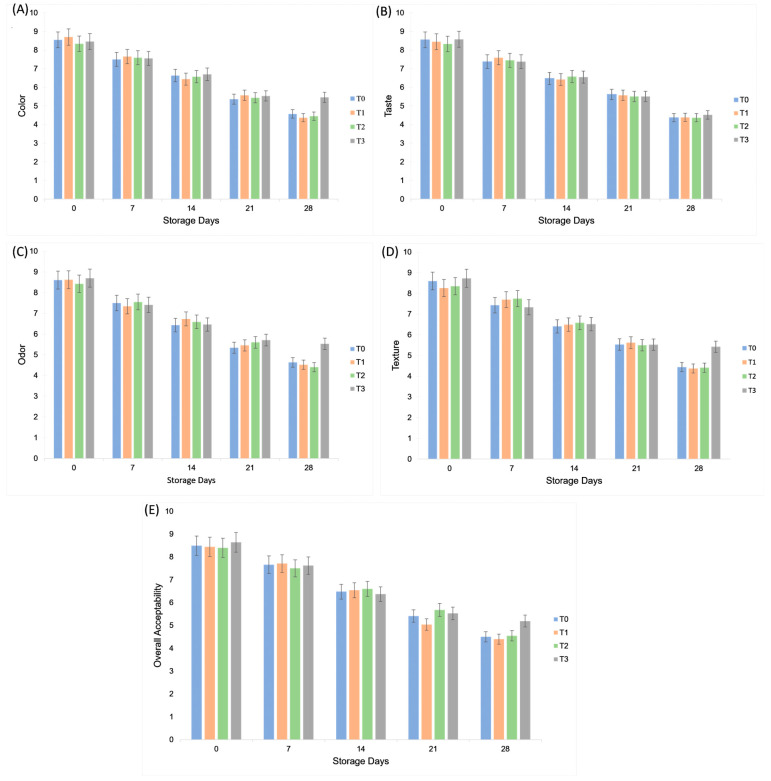
Effect of aquafaba and gum tragacanth on sensory parameters (**A**) color; (**B**) taste; (**C**) odor; (**D**) texture; (**E**) overall acceptability of plant-based mayonnaise during 28 days of refrigerated storage. Values represent mean ± SD from 12 trained panelists, evaluated in duplicate sessions.

Sensory evaluation of plant-based mayonnaise samples was conducted based on color, taste, odor, texture, and overall acceptability across 0 to 28 days of storage. For all parameters, the best scores were observed on day 0, with a gradual decline over time. Among the treatments, T_3_ consistently received the highest scores for all sensory attributes. Specifically, T_3_ recorded the top values for color (6.74 ± 1.56 ^a^), taste (6.69 ± 1.54 ^a^), odor (6.74 ± 1.43 ^a^), texture (6.68 ± 1.48 ^a^), and overall acceptability (6.64 ± 1.47 ^a^). The highest individual scores were observed on day 0, such as for texture (8.73 ± 0.81 ^a^) and overall acceptability (8.64 ± 0.76 ^a^), whereas the lowest scores were recorded on day 28 for various treatments (*p* < 0.05). The reduction in overall acceptability shows that consumer preference declined with storage time, likely due to textural and flavor changes. He, et al. [33] reported similar results for the color, odor, taste, texture, and overall acceptability of low-fat mayonnaise [33]. Due to GA’s high antioxidant activity, the oxidative stability of plant-based mayonnaise remained unchanged at the end of the storage period, which implies acceptable quality and safety for consumption [28]. During storage, the gradual increase in peroxide value reflected the onset of lipid oxidation, a process commonly associated with the development of rancid or off-odors in emulsified products. This explains the decline in odor acceptability scores observed in later storage days, as oxidation by-products can negatively influence sensory perception. As shown in Figure 9, sensory scores for appearance, taste, texture, and overall acceptability declined during storage in all treatments. However, T_3_ consistently achieved higher scores than the control, indicating better consumer acceptance of aquafaba–gum formulations.

This study provides valuable insights into the functional, sensory, and storage properties of aquafaba and gum tragacanth stabilized mayonnaise. Future studies should be conducted on direct comparison with commercially available egg-free mayonnaise products, which would allow benchmarking of physicochemical and sensory performance. Moreover, modeled nutritional labeling or caloric analysis should also be conducted. Future studies can include instrumental volatile profiling (e.g., GC–MS) alongside sensory evaluation to better elucidate the relationship between lipid oxidation, volatile release, and odor acceptability in aquafaba-based mayonnaise.

## 3. Materials and Methods

### 3.1. Raw Materials

Chickpeas (*Cicer arietinum* L.) were purchased from a local supermarket. Gum tragacanth was purchased from a local Sargodha market. The additional ingredients, vegetable oil, sugar, white vinegar, and table salt, were purchased from a local utility store. Packaging materials, such as jars and plastic bottles, were purchased from a supermarket (Sargodha, Pakistan).

### 3.2. Process of Aquafaba Production

Dry chickpeas (250 g) were rinsed and soaked in distilled water (1:3, *w*/*v*) supplemented with 0.2% NaHCO_3_ at 85 °C for 24 h. The soaked chickpeas were pressure-cooked (75–85 kPa, 110 °C) in a 5 L heavy-duty cooker (Sargodha, Pakistan) for 120 min until softened. Post cooking, aquafaba was separated using a stainless-steel strainer and stored at 4 °C for further use.

### 3.3. Preparation of Gum Tragacanth Solution

Gum tragacanth was dissolved in distilled water at concentrations of 0.3%, 0.5%, and 1.0% (*w*/*v*) using 15, 20, and 30 mL of water, respectively, for three experimental samples. The solutions were kept at 4 °C overnight to ensure complete hydration and gel formation. Following hydration, the impurities were removed by centrifugation at 500 rpm for 20 min.

### 3.4. Preparation of Plant-Based Mayonnaise

**Figure 10:**
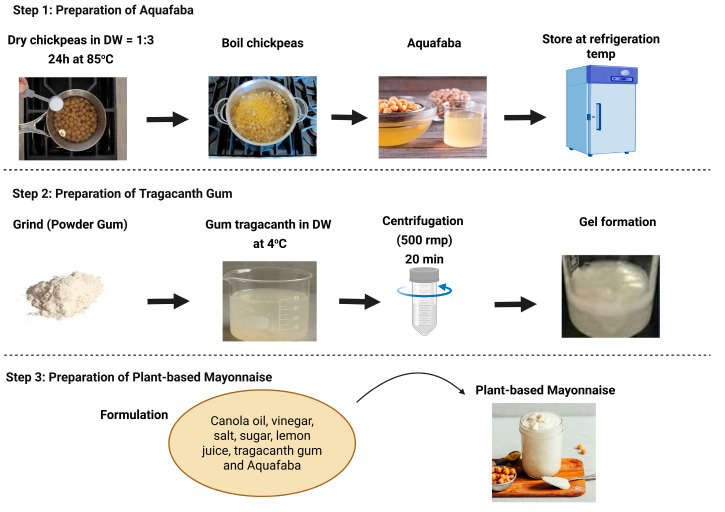
Steps of plant-based mayonnaise manufacturing with specifications.

**Figure 11:**
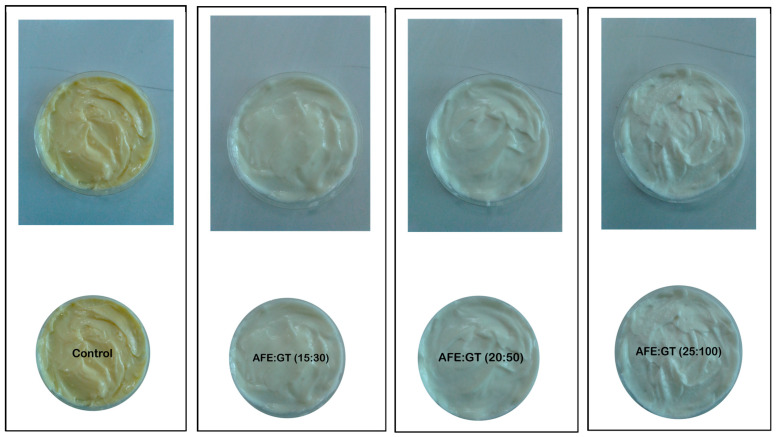
Image showing the control (egg mayonnaise), AFE:GT (15% aquafaba and 30% gum tragacanth, AFE:GT (20% aquafaba and 50% gum tragacanth, AFE:GT (25% aquafaba and 100% gum tragacanth).

The control mayonnaise (reference sample) was formulated using 80% canola oil, 15 g egg yolk, 4 mL vinegar, 0.5% sugar, and 0.5% salt (*w*/*w*) content. The control was prepared by mixing the egg yolk, sugar, salt, and vinegar for 2 min, followed by the gradual addition of oil dropwise while blending with a high-speed blender (WestPoint^®^ Professional Hand Blender, 220–240 V, 50–60 Hz, 600 Watts, WF-9714, Sargodha, Pakistan) for 5–6 min. Three samples of plant-based mayonnaise were prepared according to the method described by Raikos, et al. [34], with several modifications. In plant-based formulations, egg yolk was replaced with aquafaba, and gum tragacanth was incorporated at varying concentrations for emulsion stabilization. The plant-based mayonnaise formulations comprised canola oil, aquafaba, vinegar, sugar, salt, and gum tragacanth based on weight percentages. The formulation (%) of the control and plant-based mayonnaise is given in Table 1. Canola oil was chosen as the oil phase because of their widespread availability, neutral flavor profile, and frequent use in commercial mayonnaise formulations. It also provides a balanced fatty acid composition, making them suitable for developing plant-based emulsions. Other oils (e.g., olive, sunflower) were not included to avoid introducing strong flavors or compositional variability that could confound the effects of aquafaba and gum tragacanth. Thus, the primary controlled variables were oil type, processing conditions, and storage environment, while the manipulated variables were the aquafaba-to-oil ratio and gum tragacanth concentration. For plant-based samples, aquafaba was first mixed with sugar, salt, and vinegar for 2 min, followed by the gradual addition of oil under continuous high-speed blending for 3 min, forming a foamy and creamy emulsion. Each mayonnaise batch (300 g) was aliquoted into polyethylene bags, sealed, and stored at 4 °C for 28 days. Physicochemical properties, storage stability, and textural characteristics were evaluated at weekly intervals and compared with those of the control. At each storage day, three aliquots were taken from the same batch and analyzed in triplicate. Thus, replication in this study represents repeated measurements (technical replicates) from one biological batch per treatment. This design is standard for storage stability experiments, as it ensures that observed changes reflect the effect of storage rather than batch-to-batch variation. The steps and visual representation of control and plant-based mayonnaise manufacturing with specifications have been shown in Figure 10 and Figure 11.

### 3.5. Determination of Total Phenolic Content (TPC) and Total Flavonoid Content (TFC) in Aquafaba Extracts

The total phenolic content (TPC) and total flavonoid content (TFC) of aquafaba extracts (AFE) were determined using colorimetric methods. TPC was evaluated using the Folin–Ciocalteu method Singleton, et al. [35] with minor modifications. Gallic acid was used as the standard, and calibration solutions were prepared at concentrations of 25, 50, 75, and 100 μg/mL in ethanol. For the analysis, 5 mL of 10% Folin–Ciocalteu reagent and 4 mL of 7% sodium carbonate were added to 1 mL of the sample or standard solution, and the final volume was adjusted to 10 mL. The mixtures were incubated at 50 °C for 30 min in a water bath (WiseBath® Fuzzy Control System, model Wisd-23; DAIHAN Scientific Co., Ltd., Wonju, Republic of Korea), and the absorbance was measured at 760 nm using a UV-1100 spectrophotometer (MC, 220 V, 50 Hz). TPC was expressed as milligrams of gallic acid equivalents per gram of dry weight (mg GAE/g DW). TFC was determined using the aluminum chloride colorimetric method with Trolox as the standard. Standard solutions were prepared at 0.25, 0.5, 0.75, and 1 mg/mL in ethanol. Briefly, 1 mL of the sample or standard was mixed with 4 mL distilled water, followed by the addition of 0.3 mL 5% NaNO_2_, 0.3 mL 10% AlCl_3_ after 5 min, and 2 mL 1 M NaOH after another 6 min. The volume was adjusted to 10 mL with distilled water, and the absorbance was measured at 510 nm. TFC was calculated from the calibration curve and expressed as milligrams of Trolox equivalents per gram of dry weight (mg TE/g DW). All measurements were performed in triplicates.

### 3.6. Anti-Oxidant Activity

The antioxidant activity of plant-based mayonnaise samples was evaluated using the phosphomolybdate and DPPH assays with slight modifications from Prieto, et al. [36]. For the phosphomolybdate assay, an antioxidant reagent was prepared by mixing 0.6 M H_2_SO_4_, 28 mM sodium phosphate, and 4 mM ammonium molybdate. Approximately 5 g of each mayonnaise sample was diluted with 45 mL of chloroform, and 0.4 mL of the diluted sample was mixed with 4 mL of the antioxidant reagent in test tubes, incubated in a water bath at 85 °C for 90 min, and the absorbance was measured at 690 nm using a UV–Vis spectrophotometer (model SCI-UV1100; SCILOGEX, LLC, Rocky Hill, CT, USA; 220 V, 50 Hz). For the DPPH assay, 10 g of the mayonnaise sample was diluted with 2.5 mL of chloroform and adjusted to pH 4.0 with 1 M HCl. Samples were incubated at 45 °C for 10 min, and peptide extracts were obtained by centrifugation at 10,000 rpm at 4 °C for 10 min. The extracted peptide solution (1 mL) was mixed with 4 mL of distilled water and 1 mL of DPPH solution. A control solution was prepared without the sample. Absorbance was recorded at 517 nm, and the antioxidant activity (%) was calculated using the following formula:

Anti-oxidant (%) = control sample − extracted sample ÷ control sample × 100

Scavenging rate (%) = [1 − (A/A0)] × 100

where, A = Absorbance of the sample A0 = Absorbance of blank

### 3.7. Physicochemical Analysis

The physicochemical parameters of plant-based mayonnaise, such as moisture, fat, ash, crude fiber, crude protein, and carbohydrate content (nitrogen-free extract, NFE), were determined following standard methods described by the Association of Official Analytical Chemists [37]. The moisture content in the plant-based mayonnaise treatments was analyzed using a hot air oven (Model: ED-115) Binder, Germany. Physicochemical analysis of plant-based mayonnaise was performed using standard methods (AOAC Method No. 930.15), ash (AOAC Method No. 942.05), and acidity (AOAC Method No. 947.05). Crude protein content was determined using the Kjeldahl method. Briefly, approximately 1 g of mayonnaise sample was digested with 20 mL of concentrated H_2_SO_4_ and 5 g of digestion mixture (K_2_SO_4_ 90%, CuSO_4_ 7%, FeSO_4_ 3%) at 400 °C until a clear light green solution was obtained. The digested sample was diluted and distilled with 40% NaOH in a Kjeldahl apparatus, and the released ammonia was trapped in 2% boric acid containing a methyl red indicator. Titration was performed using 0.05 N H_2_SO_4_ until a golden-brown endpoint was reached. The fat content of plant-based mayonnaise was evaluated using the Gerber method with some modifications, as described by Marshall [38]. The Gerber method is a volumetric method that employs chemical reagents, such as sulfuric acid, to break down the emulsion and separate the fat. A special flask, known as a butyrometer, was used to measure the fat content. Ten milliliters of mayonnaise were transferred to a butyrometer, followed by the addition of 10 mL concentrated H_2_SO_4_ and 3–4 mL isoamyl alcohol. The mixture was carefully inverted for complete mixing and placed in a water bath at 65 °C for 30 min. After heating, the butyrometer was centrifuged at 1100 rpm for 5 min in a Gerber centrifuge. The crude fat content (%) was directly read from butyrometer graduations. The crude fiber content was determined using acid and alkali digestion. Briefly, 2 g of fat- and moisture-free sample was boiled with 150 mL of 1.25% H_2_SO_4_ for 30 min. After filtration and thorough washing, the residue was boiled again with 150 mL of 1.25% NaOH solution for 30 min, followed by filtration and washing with hot, distilled water. The residue was dried at 70–80 °C overnight, weighed, and incinerated in a muffle furnace at 550 °C for 5–6 h. The pH of the plant-based mayonnaise samples was measured using a digital pH meter (Model: pH N-81, Tacussel Electronique, Villeurbanne, France) according to AOAC method 981.12 [39]. The peroxide value (PV) was determined according to the method described by Shantha and Decker [40], based on the formation of an iron–thiocyanate complex. All analyses were performed at 0, 7, 14, 21, and 28 d of storage.

### 3.8. Creaming Index Analysis

The creaming index (CI) of mayonnaise samples was assessed according to the method described by Shamooshaki, et al. [41]. Fifty milliliters of each freshly prepared sample was placed in cylindrical glass tubes, sealed, and stored at 4 °C for 28 days. The creaming index was calculated using the following equation:

CI (%) = H2 ÷ H1 × 100

where

H1 = Initial height of emulsion

H2 = Alteration in bottom serum height during time of storage

### 3.9. Physical and Heat Stability (%)

The physical and thermal stabilities of the mayonnaise samples were evaluated as described by Mun, et al. [42] with slight modifications. For physical stability, approximately 15 g of each sample was placed in 15 mL centrifuge tubes and centrifuged at 4000 rpm for 45 min at room temperature. The separated oil layer was removed, and the precipitated fraction was weighed. For heat stability, 10 g of mayonnaise was heated at 80 °C in a water bath (WiseBath Fuzzy Control System, Wisd-23) for 30 min, followed by centrifugation at 4000 rpm for 45 min. Physical and heat stability (%) were calculated using the following formula:

Stability (%) = F2 ÷ F1 × 100

F1 = Original samples weight (g)

F2 = Precipitated fraction weight (g)

### 3.10. Microbiological Analysis

The total mold count was determined by preparing a potato dextrose agar (PDA) medium, where 10 g PDA was dissolved in 250 mL normal saline and sterilized along with Petri dishes and test tubes in an autoclave for 30 min. Mayonnaise samples (5 g) were diluted with 45 mL of chloroform, and serial dilutions were prepared by adding 1 mL of the sample to 9 mL of normal saline, followed by a second dilution under sterile conditions in a laminar flow hood. Approximately 10 mL of warm PDA was poured into Petri dishes and allowed to solidify. Diluted and double-diluted samples (2–3 mL) were inoculated onto PDA plates, dried, and incubated at 35 °C for 48 h. Mold colonies were counted manually, and the total mold count (CFU/mL) was calculated using the following formula:

Total Mold Count = Average number of colonies × dilution factor ÷ volume Mold count expressed as CFU/mL

### 3.11. Viscosity Analysis

The viscosity of the mayonnaise samples was measured using a digital viscometer (Spindle R 4; AMETEK Brookfield, Middleboro, MA, USA) at 35–36 °C and 1500 rpm for 30 s, following the method described by Sindhu, et al. [43]. The spindle was immersed in the sample, and the viscosity readings were recorded directly.

### 3.12. Texture Analysis

The textural properties of plant-based mayonnaise were evaluated using a TA.XT Plus texture analyzer equipped with a P/75 compression plate (75-mm) probe (Stable Micro Systems Ltd., Godalming, Surrey, UK), following the method described by Raikos, Hayes and Ni [34]. Approximately 80 g of the sample was placed in a cylinder, and the probe was penetrated to a depth of 40 mm. Firmness was measured as the maximum load during the first compression cycle, which represents the resistance to probe penetration.

### 3.13. Fourier-Transform Infrared Spectroscopy (FTIR) Analysis

The control and plant-based mayonnaise samples were stored at 4 °C prior to analysis. Approximately 5 g of each sample was freeze-dried in Eppendorf tubes for 24 h, followed by thawing. FTIR analysis was performed to determine the functional groups following the method described by Chippie, et al. [44] with slight modifications. An IR Prestige-21 spectrometer (Shimadzu, Japan) equipped with an AIM-8800 automatic infrared microscope (Shimadzu Corporation, Kyoto, Japan) and Spectrum for Windows IR Solution software Version 1.04 were used. Prior to the measurements, the desiccator was operated for 24 h to prevent moisture interference. The scanning parameters were set in the range of 400–4000 cm^−1^ with a resolution of 1 cm^−1^. Semi-solid samples were analyzed using a liquid NaCl cell assembly, where a drop of the sample was placed on the cell, and scanning was performed using the diffuse reflectance technique. IR light was passed through the sample, and the spectra were collected after ten successive scans. Functional groups were identified based on absorption peaks, and spectra from plant-based mayonnaise treatments were compared with those of the control sample.

### 3.14. Sensory Evaluation

The sensory attributes of plant-based mayonnaise, including color, taste, odor, texture, spreadability, and overall acceptability, were evaluated by a trained sensory panel following the 9-point hedonic scale described by Herald, et al. [45]. The panel consisted of 12 trained individuals (6 male, 6 female) aged 22–45 years, with prior experience in evaluating emulsion-based products. All panelists were non-smokers and regular consumers of mayonnaise-type condiments. Prior to formal evaluation, panelists underwent two 1 h training sessions to familiarize them with sensory descriptors and scoring procedures. Mayonnaise samples were spread on bread slices to assess spreadability and presented in randomized order under standardized lighting and serving conditions. Water was provided for palate cleansing between samples. Panelists rated each attribute on a scale from 1 (extremely dislike) to 9 (extremely like), and scores were averaged to assess sensory performance.

### 3.15. Statistical Analysis

All obtained data were statistically analyzed using two-way analysis of variance (ANOVA) in IBM SPSS Statistics version 26. The effectiveness and acceptability of the plant-based mayonnaise samples were evaluated, following Steel and Torrie [46]. Fisher’s least significant difference (LSD) post-hoc test was applied for pairwise comparisons. Results are presented as mean ± standard deviation (SD). All analyses were conducted with three biological replicates; each measured in triplicate (technical replicates). Different lowercase letters in tables and figures denote statistically significant differences among means within the same parameter according to Fisher’s LSD test (*p < 0.05*).

## 4. Conclusions

This study demonstrated that aquafaba, in combination with gum tragacanth, can successfully replace egg yolk in mayonnaise, producing emulsions with desirable stability, antioxidant activity, and sensory acceptability. The formulation with 25% aquafaba and 1% gum tragacanth showed the best balance of texture, viscosity, oxidative stability, and consumer preference. Beyond functional quality, these findings highlight the broader potential of aquafaba–gum tragacanth systems as clean-label, cholesterol-free, and allergen-friendly alternatives to conventional mayonnaise. Such formulations align with current consumer trends toward vegan, plant-based, and sustainable foods. Future research should address the extended shelf-life studies, rheological and lipid oxidation profiling, and consumer-based sensory trials to strengthen industrial applicability. Overall, aquafaba and gum tragacanth represent a promising platform for developing novel plant-based condiments with both scientific and commercial relevance.

## Figures and Tables

**Table 1:** The formulation (%) of the traditional mayonnaise (control) and plant-based mayonnaise.

	Samples			
Ingredients (%)	Control (T_0_)	GT 30% (T_1_)	GT 50% (T_2_)	GT 100% (T_3_)
Canola oil	80	80	75	70
Egg	15	-	-	-
Vinegar	4	3	3	3
Salt	0.5	0.5	0.5	0.5
Sugar	0.5	0.5	0.5	0.5
Lemon Juice	1	1	1	1
Tragacanth Gum	-	0.3	0.5	1
Aquafaba	-	15	20	25

where T_0_ represents aquafaba to oil ratio: (0:80) and 15% egg yolk, T_1_ represents aquafaba to oil ratio: (15:80) and 0.3% gum tragacanth, T_2_ represents aquafaba to oil ratio: (20:75) and 0.5% gum tragacanth, T_3_ represents aquafaba to oil ratio: (25:70) and 1% gum tragacanth.

## Data Availability

All the original data presented in this research are included in the article. Further information is available from the first author or the corresponding author upon reasonable request.

## Supplementary Materials

The following supporting information can be downloaded at: https://www.mdpi.com/article/10.3390/molecules30173511/s1, 1. Raw Materials; 2. Preparation of Aquafaba; 3. Preparation of Gum Tragacanth (GT) Solution; 4. Preparation of Control Mayonnaise (T_0_); 5. Preparation of Plant-based Mayonnaise (T_1_-T_3_); 6. Storage and Sampling; Table S1: Analysis of Variance for TPC (mg GAE/g); Table S2: Analysis of Variance for TFC (mg TE/g); Table S3: Analysis of Variance for Moisture Content (%); Table S4: Fisher LSD for Moisture Content; Table S5: Analysis of Variance for Fat Content (%); Table S6: Fisher LSD for Fat Content (%); Table S7: Analysis of Variance for Protein Content (%); Table S8: Fisher LSD for Protein Content (%); Table S9: Analysis of Variance for Fiber Content (%); Table S10: Fisher LSD for Fiber Content (%); Table S11: Analysis of Variance for Nitrogen-Free Extract (%); Table S12: Fisher LSD for Nitrogen-Free Extract (%); Table S13: Analysis of Variance for pH; Table S14: Fisher LSD for pH; Table S15: Analysis of Variance for Acidity (%); Table S16: Fisher LSD for Acidity (%); Table S17: Analysis of Variance for Peroxide Value (meq/kg); Table S18: Fisher LSD for Peroxide Value (meq/kg); Table S19: Analysis of Variance for Creaming Index; Table S20: Fisher LSD for Creaming Index; Table S21: Analysis of Variance for Physical Stability; Table S22: Fisher LSD for Physical Stability; Table S23: Analysis of Variance for Heat Stability; Table S24: Fisher LSD for Heat Stability; Table S25: Analysis of Variance for Phosphomolybdate Assay; Table S26: Fisher LSD for Phosphomolybdate Assay; Table S27: Analysis of Variance for DPPH Assay; Table S28: Fisher LSD for DPPH Assay; Table S29: Analysis of Variance for Mold Count; Table S30: Fisher LSD for Mold Count; Table S31: Analysis of Variance for Viscosity; Table S32: Fisher LSD for Viscosity; Table S33: Analysis of Variance for Firmness; Table S34: Fisher LSD for Firmness; Table S35: Analysis of Variance for Color; Table S36: Fisher LSD for Color; Table S37: Analysis of Variance for Taste; Table S38: Fisher LSD for Taste; Table S39: Analysis of Variance for Odor; Table S40: Fisher LSD for Odor; Table S41: Analysis of Variance for Texture; Table S42: Fisher LSD for Texture; Table S43: Analysis of Variance for Overall Acceptability; Table S44: Fisher LSD for Overall Acceptability.
