# Supplementary material for: Development and Evaluation of Egg-Free Mayonnaise Stabilized with Aquafaba and Gum Tragacanth: Functional, Sensory, and Storage Properties
Source: Molecules. 2025 Aug 27;30(17):3511. doi: 10.3390/molecules30173511 (PMC12430565; doi:10.3390/molecules30173511)
Supplement: Supplementary file 1 [file molecules-30-03511-s001.zip › molecules-3823207-supplementary.pdf]

Evaluation of Tragacanth Gum as a Potential Stabilizer in the Development of Plant-based Mayonnaise

Bakhtawar Shafique [1,2], Mian Anjum Murtaza [1], Muhammad Salman Farid [3], Kashif Ameer [1], Muhammad Imran Hussain [4], Monika Sienkiewicz [5], Anna Lichota [5*] and Łukasz Łopusiewicz [3,6*]

[1] Institute of Food Science and Nutrition, University of Sargodha, Sargodha 40100, Pakistan; bakhtawarshafique111@gmail.com (B.S.); anjum.murtaza@uos.edu.pk (M.A.M); kashif.ameer@uos.edu.pk (K.A.)
[2] College of Food Science and Technology, Shanghai Ocean University, Shanghai 201306, China; bakhtawarshafique111@gmail.com (B.S.)
[3] Institute of Pharmacy, Department Pharmaceutical Biology, Greifswald University, Friedrich-Ludwig-Jahn-Str. 17, 17489 Greifswald, Germany; salmanfarid9187@gmail.com (M.S.F.)
[4] Department of Human Nutrition and Dietetics, Rashid Latif Khan University, Lahore, Pakistan; Imran.hussain@rlku.edu.pk (M.I.H.)
[5] Department of Pharmaceutical Microbiology and Microbiological Diagnostics, Medical University of Lodz, 90-151 Łódź, Poland; monika.sienkiewicz@umed.lodz.pl (M.S.); anna.lichota@umed.lodz.pl (A.L.)
[6] School of Medical & Health Sciences, Vizja University, 59 Okopowa Str. Warsaw, 01-043, Poland; l.lopusiewicz@vizja.pl (Ł.Ł.)

* Correspondence: l.lopusiewicz@vizja.pl; anna.lichota@umed.lodz.pl

1. Raw Materials

Chickpeas (*Cicer arietinum* L.) were purchased from a local supermarket in Sargodha, Pakistan, while gum tragacanth (GT) was sourced from a local herbal market. Additional ingredients, including canola oil, white vinegar (5% acetic acid), table salt, granulated sugar, and lemon juice, were obtained from a local utility store, and packaging materials such as polyethylene bags, jars, and plastic bottles were purchased from a supermarket.

2. Preparation of Aquafaba

Aquafaba was obtained by cooking chickpeas under controlled conditions. First, 250 g of dry chickpeas were rinsed thoroughly and soaked in distilled water (1:3, w/v) supplemented with 0.2% $NaHCO_3$ at 85 °C for 24 h. The soaked chickpeas were then pressure-cooked in a 5 L heavy-duty cooker at 75–85 kPa and 110 °C for 120 min until softened. After cooking, the viscous cooking liquid (aquafaba) was carefully separated using a stainless-steel strainer, cooled to room temperature, and stored at 4 °C in sterile glass containers for up to 48 h until further use.

3. Preparation of Gum Tragacanth (GT) Solution

Gum tragacanth solutions were prepared at three concentrations: 0.3%, 0.5%, and 1.0% (w/v). For each concentration, GT powder was dispersed in distilled water with continuous stirring until homogeneity was achieved. The dispersions were then refrigerated at 4 °C overnight to allow complete hydration and gel formation. Following hydration, the solutions were centrifuged at 500 rpm for 20

min to remove any insoluble impurities. The resulting clear solutions were collected and used directly in the preparation of plant-based mayonnaise.

4. Preparation of Control Mayonnaise (T_0)

The control mayonnaise (T_0) was prepared using 80% canola oil, 15% egg yolk, 4% vinegar, 0.5% sugar, 0.5% salt, and 1% lemon juice (w/w). Egg yolk, vinegar, sugar, salt, and lemon juice were blended for 2 min, followed by the addition of canola oil under continuous mixing with a high-speed hand blender (WestPoint® WF-9714, 600 W, 220–240 V, 50–60 Hz) for 5–6 min, yielding a smooth and stable emulsion.

5. Preparation of Plant-based Mayonnaise (T_1-T_3)

Plant-based mayonnaise samples (T_1–T_3) were prepared by replacing egg yolk with aquafaba and incorporating gum tragacanth at different concentrations. In each case, aquafaba was mixed with vinegar, sugar, salt, and lemon juice for 2 min, after which the respective GT solution was added. Canola oil was then incorporated under continuous blending for 3–4 min, producing a creamy and homogeneous emulsion. The formulations differed in aquafaba-to-oil ratios and GT levels: T_1 contained 15% aquafaba, 80% oil, and 0.3% GT; T_2 contained 20% aquafaba, 75% oil, and 0.5% GT; and T_3 contained 25% aquafaba, 70% oil, and 1.0% GT. Each batch (300 g) of mayonnaise was packed into sterile polyethylene bags, sealed, and stored at 4 °C for up to 28 days.

6. Storage and Sampling

During storage, physicochemical, microbiological, antioxidant, and sensory analyses were performed on days 0, 7, 14, 21, and 28. At each time point, three aliquots were withdrawn from the same batch and analyzed in triplicate, meaning that replication in this study represented technical replicates obtained from one biological batch per treatment. This design ensured that the observed changes reflected the effect of storage conditions rather than batch-to-batch variation.

Table S1. Analysis of Variance for TPC (mg GAE/g)

Source of Variation	SS	df	MS	F	P-value	F crit
Aquafaba Extract	25697.4	4	6424.36	431.6	0.050395*	2.003582
Within	148.8	10	14.88			
Total	25846.3	14				

*p<0.05; **p<0.01; ***p<0.001; ns: non-significant

Table S2. Analysis of Variance for TFC (mg TE/g)

Source of Variation	SS	df	MS	F	P-value	F crit
**Aquafaba Extract**	25176.1	4	6294.03	1782.23	0.050484*	2.004395
**Within**	35.3	10	3.53			
**Total**	25211.4	14				

*p<0.05; **p<0.01; ***p<0.001; ns: non-significant

Table S3. Analysis of Variance for Moisture Content (%)

Source of Variation	SS	df	MS	F	P-value	F crit
**Treatment**	106.9264	3	35.642133	12.432081	6.94E-06	2.838745
**Storage Interval**	29.47076	4	7.3676892	2.5698716	0.050482*	2.605975
**T*S**	19.58388	12	1.6319903	0.569243	0.050354*	2.003459
**Within**	114.6779	40	2.8669483			
**Total**	270.659	59				

*p<0.05; **p<0.01; ***p<0.001; ns: non-significant

Table S4. Fisher LSD for Moisture Content

Storage Days	Treatments				Mean±SE
	T_0	T_1	T_2	T_3	
**0**	32.72±1.3 ^{a}	30.3±1.44 ^{abc}	30.86±1.64 ^{abc}	29.86±2.34 ^{abc}	**30.94±1.86 ^{a}**
**7**	32.04±1.31^{ab}	31.08±2.31 ^{abc}	29.65±1.33 ^{abc}	28.71±2.3 ^{abc}	**30.37±2.09 ^{ab}**

**14**	30.46±1.35 abc	30.64±2.01 abc	29.13±1.1 abc	28.16±2.1 abc	**29.66±1.79 ab**
**21**	31.72±1.3 abc	30.63±2.1 abc	28.68±1.23 abc	27.63±2.1 abc	**29.6±2.23 ab**
**28**	31.8±1.29 abc	29.67±1.2 abc	27.39±1.47 bc	26.63±1.54 bc	**28.87±2.42 b**
**Mean±SE**	**31.75±1.34 a**	**30.46±1.64 ab**	**29.14±1.65 bc**	**28.2±2.09 c**	

T0: Control

T1: 15% Aquafaba Extract + 30% Gum Tragacanth

T2: 20% Aquafaba Extract + 50% Gum Tragacanth

T3: 25% Aquafaba Extract + 100% Gum Tragacanth

Means sharing different letter are significantly different ($p < 0.05$)

Table S5. Analysis of Variance for Fat Content (%)

Source of Variation	SS	df	MS	F	P-value	F crit
**Treatment**	771.902	3	257.3007	102.366605	8.2765E-19	2.838745
**Storage Interval**	24.68436	4	6.171089	2.45515654	0.06122849^{ns}	2.605975
**T*S**	12.47991	12	1.039993	0.41375911	0.94931334^{ns}	2.003459
**Within**	100.5409	40	2.513522			
**Total**	909.6072	59				

*p<0.05; **p<0.01; ***p<0.001; ns: non-significant

Table S6. Fisher LSD for Fat Content (%)

Storage Days	Treatments				Mean±SE
	**T_0**	**T_1**	**T_2**	**T_3**	
**0**	67.03±1.55 bc	61.89±1.68 efgh	60.4±1.5 ghijk	57.97±1.6 k	**61.82±3.72 b**
**7**	67.98±1.74 ab	62.41±1.44 defg	61.12±1.84 efghij	58.51±1.6 jk	**62.5±3.88 ab**
**14**	69.81±1.7 a	63.32±1.29 def	60.74±1.76 fghij	59.04±1.6 ijk	**63.23±4.49 a**

**21**	69.21±1.7 [ab]	63.66±1.44 [de]	61.03±1.76 [efghij]	59.58±1.6 [hijk]	**63.37±4.08 [a]**
**28**	68.64±1.68 [ab]	64.65±1.46 [cd]	61.21±1.6 [efghi]	60.11±1.6 [ghijk]	**63.65±3.73 [a]**
**Mean±SE**	**68.53±1.73 [a]**	**63.19±1.59 [b]**	**60.9±1.47 [c]**	**59.04±1.56 [d]**	

T0: Control

T1: 15% Aquafaba Extract + 30% Gum Tragacanth

T2: 20% Aquafaba Extract + 50% Gum Tragacanth

T3: 25% Aquafaba Extract + 100% Gum Tragacanth

Means sharing different letter are significantly different ($p < 0.05$)

Table S7. Analysis of Variance for Protein Content (%)

Source of Variation	SS	df	MS	F	P-value	F crit
**Treatment**	239.1533	3	79.7177774	19.8699579	4.802E-08	2.838745
**Storage Interval**	18.67057	4	4.66764352	1.16342782	0.3413604[ns]	2.605975
**T*S**	49.93786	12	4.16148874	1.03726683	0.4356194[ns]	2.003459
**Within**	160.479	40	4.01197515			
**Total**	468.2408	59				

*p<0.05; **p<0.01; ***p<0.001; ns: non-significant

Table S8. Fisher LSD for Protein Content (%)

Storage Days	Treatments				Mean±SE
	**T_0**	**T_1**	**T_2**	**T_3**	
**0**	12.82±2.15 [h]	15.69±1.86 [cdefgh]	16.94±2.1 [bcdef]	18.85±2.1 [bc]	**16.08±2.88 [a]**
**7**	13.36±2.15 [gh]	16.28±2.1 [bcdefg]	22.28±1.16 [a]	18.69±2 [bc]	**17.65±3.78 [a]**
**14**	13.64±1.77 [fgh]	16.71±2.1 [bcdef]	17.56±2.1 [bcde]	18.66±2.15 [bc]	**16.64±2.61 [a]**
**21**	14.41±2.1 [efgh]	17.13±2.1 [bcde]	17.87±2.1 [bcd]	18.67±1.8 [bc]	**17.02±2.41 [a]**

**28**	14.75±1.86 [defgh]	17.55±2.1 [bcde]	18.18±2.1 [bc]	19.13±1.95 [a]	**17.4±2.41 [a]**
**Mean±SE**	**13.8±1.85 [c]**	**16.67±1.86 [b]**	**18.57±2.57 [a]**	**18.8±1.7 [a]**	

T0: Control

T1: 15% Aquafaba Extract + 30% Gum Tragacanth

T2: 20% Aquafaba Extract + 50% Gum Tragacanth

T3: 25% Aquafaba Extract + 100% Gum Tragacanth

Means sharing different letter are significantly different ($p < 0.05$)

Table S9. Analysis of Variance for Fiber Content (%)

Source of Variation	SS	df	MS	F	P-value	F crit
**Treatment**	60.48178	3	20.16059	4.779204	0.006128**	2.838745
**Storage Interval**	9.315532	4	2.328883	0.552077	0.698599ns	2.605975
**T*S**	0.756708	12	0.063059	0.014949	1ns	2.003459
**Within**	168.736	40	4.2184			
**Total**	239.29	59				

*p<0.05; **p<0.01; ***p<0.001; ns: non-significant

Table S10. Fisher LSD for Fiber Content (%)

Storage Days	Treatments				Mean±SE
	T_0	T_1	T_2	T_3	
**0**	9.65±1.97 [ab]	8.57±2.02 [ab]	7.8±2.17 [ab]	6.71±2.19 [b]	**8.18±2.11 [a]**
**7**	9.61±1.96 [ab]	8.67±1.95 [ab]	7.69±2.06 [ab]	6.84±1.87 [b]	**8.2±1.99 [a]**
**14**	9.82±1.97 [ab]	8.76±1.91 [ab]	8.4±2.06 [ab]	7.5±2.18 [ab]	**8.62±1.94 [a]**
**21**	10.54±2.36 [a]	9.42±2.28 [ab]	8.58±2 [ab]	7.64±1.96 [ab]	**9.04±2.15 [a]**
**28**	10.43±2.25 [a]	8.7±2.18 [ab]	8.7±1.8 [ab]	7.68±1.81 [ab]	**9.1±2.03 [a]**

Mean±SE	10.01±1.83 ^{a}	9±1.8 ^{a}	8.23±1.76 ^{bc}	7.27±1.75 ^{c}	

T0: Control

T1: 15% Aquafaba Extract + 30% Gum Tragacanth

T2: 20% Aquafaba Extract + 50% Gum Tragacanth

T3: 25% Aquafaba Extract + 100% Gum Tragacanth

Means sharing different letter are significantly different (p < 0.05)

Table S11. Analysis of Variance for Nitrogen-Free Extract (%)

Source of Variation	SS	df	MS	F	P-value	F crit
**Treatment**	133.353	3	44.45101	11.03065	2.06E-05	2.838745
**Storage Interval**	1.660365	4	0.415091	0.103006	0.980804^{ns}	2.605975
**T*S**	0.286087	12	0.023841	0.005916	1^{ns}	2.003459
**Within**	161.1909	40	4.029773			
**Total**	296.4904	59				

*p<0.05; **p<0.01; ***p<0.001; ns: non-significant

Table S12. Fisher LSD for Nitrogen-Free Extract (%)

Storage Days	Treatments				Mean±SE
	**T_0**	**T_1**	**T_2**	**T_3**	
**0**	11.19±2.09 ^{abcd}	8.26±1.99 ^{de}	8.28±1.99 ^{cde}	7.5±1.99 ^{e}	**8.81±2.26 ^{a}**
**7**	11.38±2.09 ^{abcd}	8.31±1.99 ^{cde}	8.41±1.99 ^{cde}	7.58±1.99 ^{e}	**8.92±2.29 ^{a}**
**14**	11.58±2.09 ^{abc}	8.36±1.99 ^{cde}	8.54±1.99 ^{bcde}	7.65±1.99 ^{e}	**9.03±2.33 ^{a}**
**21**	11.77±2.09 ^{ab}	8.41±1.99 ^{cde}	8.67±1.99 ^{abcde}	7.72±1.99 ^{e}	**9.14±2.36 ^{a}**
**28**	11.89±1.99 ^{a}	8.46±1.99 ^{bcde}	8.8±1.99 ^{abcde}	7.79±1.99 ^{e}	**9.24±2.36 ^{a}**
**Mean±SE**	**11.56±1.77 ^{a}**	**8.36±1.68 ^{b}**	**8.54±1.69 ^{b}**	**7.65±1.68 ^{b}**	

T0: Control

T1: 15% Aquafaba Extract + 30% Gum Tragacanth

T2: 20% Aquafaba Extract + 50% Gum Tragacanth

T3: 25% Aquafaba Extract + 100% Gum Tragacanth

Means sharing different letter are significantly different ($p < 0.05$)

Table S13. Analysis of Variance for pH

Source of Variation	SS	df	MS	F	P-value	F crit
**Treatment**	2.869601	3	0.956534	73.95983	2.27E-16	2.838745
**Storage Interval**	1.137357	4	0.284339	21.98531	1.17E-09	2.605975
**T*S**	0.146903	12	0.012242	0.946554	0.005128**	2.003459
**Within**	0.517326	40	0.012933			
**Total**	4.671187	59				

*p<0.05; **p<0.01; ***p<0.001; ns: non-significant

Table S14. Fisher LSD for pH

Storage Days	Treatments				Mean±SE
	T_0	T_1	T_2	T_3	
**0**	4.3±0.13 ^{a}	3.96±0.12 ^{cd}	3.67±0.08 ^{efghi}	3.83±0.11 ^{de}	**3.94±0.26 ^{a}**
**7**	4.36±0.1 ^{a}	3.78±0.09 ^{def}	3.65±0.13 ^{efghi}	3.7±0.1 ^{efg}	**3.87±0.31 ^{ab}**
**14**	4.27±0.31 ^{ab}	3.67±0.1 ^{efgh}	3.57±0.11 ^{fghi}	3.58±0.1 ^{fghi}	**3.77±0.34 ^{bc}**
**21**	4.06±0.19 ^{bc}	3.64±0.1 ^{efghi}	3.51±0.11 ^{ghij}	3.45±0.1 ^{ij}	**3.67±0.27 ^{c}**
**28**	3.85±0.1 ^{cde}	3.47±0.11 ^{hij}	3.45±0.11 ^{ij}	3.33±0.1 ^{j}	**3.53±0.22 ^{d}**
**Mean±SE**	**4.17±0.25 ^{a}**	**3.7±0.19 ^{b}**	**3.57±0.12 ^{c}**	**3.58±0.2 ^{c}**	

T0: Control

T1: 15% Aquafaba Extract + 30% Gum Tragacanth

T2: 20% Aquafaba Extract + 50% Gum Tragacanth

T3: 25% Aquafaba Extract + 100% Gum Tragacanth

Means sharing different letter are significantly different (p < 0.05)

Table S15. Analysis of Variance for Acidity (%)

Source of Variation	SS	df	MS	F	P-value	F crit
**Treatment**	11.5406	3	3.846868	189.6602	1.11E-23	2.838745
**Storage Interval**	1.028877	4	0.257219	12.68155	9.38E-07	2.605975
**T*S**	0.474361	12	0.03953	1.948932	0.057056*	2.003459
**Within**	0.811318	40	0.020283			
**Total**	13.85516	59				

*p<0.05; **p<0.01; ***p<0.001; ns: non-significant

Table S16. Fisher LSD for Acidity (%)

Storage Days	Treatments				Mean±SE
	T_0	T_1	T_2	T_3	
**0**	0.61±0.12 ^{l}	0.82±0.14 ^{jkl}	1.44±0.14 ^{efg}	1.84±0.14 ^{bc}	**1.18±0.52** ^{c}
**7**	0.73±0.15 ^{kl}	1.01±0.21 ^{ij}	1.47±0.1 ^{efg}	1.87±0.08 ^{bc}	**1.27±0.47** ^{bc}
**14**	0.8±0.16 ^{jkl}	1.18±0.2 ^{hi}	1.55±0.11 ^{def}	1.92±0.06 ^{b}	**1.36±0.45** ^{b}
**21**	0.83±0.13 ^{jkl}	1.3±0.2 ^{gh}	1.63±0.1 ^{cde}	1.75±0.09 ^{bcd}	**1.38±0.39** ^{b}
**28**	0.88±0.12 ^{jk}	1.36±0.14 ^{fgh}	1.74±0.13B ^{cd}	2.3±0.28 ^{a}	**1.57±0.57** ^{a}
**Mean±SE**	**0.77±0.15** ^{d}	**1.13±0.25** ^{c}	**1.57±0.15** ^{b}	**1.94±0.24** ^{a}	

T0: Control

T1: 15% Aquafaba Extract + 30% Gum Tragacanth

T2: 20% Aquafaba Extract + 50% Gum Tragacanth

T3: 25% Aquafaba Extract + 100% Gum Tragacanth

Means sharing different letter are significantly different (p < 0.05)

Table S17. Analysis of Variance for Peroxide Value (meq/kg)

Source of Variation	SS	df	MS	F	P-value	F crit
**Treatment**	242.3357	3	80.77857	100.058	1.24E-18	2.838745
**Storage Interval**	70.75121	4	17.6878	21.90936	1.23E-09	2.605975
**T*S**	3.598388	12	0.299866	0.371435	0.009662**	2.003459
**Within**	32.29269	40	0.807317			
**Total**	348.978	59				

*p<0.05; **p<0.01; ***p<0.001; ns: non-significant

Table S18. Fisher LSD for Peroxide Value (meq/kg)

Storage Days	Treatments				Mean±SE
	T_0	T_1	T_2	T_3	
**0**	12.35±1.11 ^{cd}	8.36±0.84 ^{hij}	7.3±0.84 ^{jkl}	6.03±0.84 ^{l}	**8.51±2.59 ^{d}**
**7**	12.6±0.76B ^{cd}	9.16±0.84 ^{gh}	8.13±0.84 ^{hij}	6.7±0.84 ^{kl}	**9.15±2.38 ^{d}**
**14**	13.22±0.84 ^{abc}	9.95±0.84 ^{fg}	8.95±0.84 ^{gh}	7.37±0.84 ^{ijkl}	**9.87±2.35 ^{c}**
**21**	13.8±0.84 ^{ab}	10.75±0.84 ^{ef}	9.78±0.84 ^{fg}	8.04±0.84 ^{hijk}	**10.59±2.3 ^{b}**
**28**	14.38±0.84 ^{a}	11.54±0.84 ^{de}	10.6±0.84 ^{ef}	8.71±0.84 ^{ghi}	**11.31±2.25 ^{a}**
**Mean±SE**	**13.27±1.08 ^{a}**	**9.95±1.36 ^{b}**	**8.95±1.4 ^{c}**	**7.37±1.21 ^{d}**	

T0: Control

T1: 15% Aquafaba Extract + 30% Gum Tragacanth

T2: 20% Aquafaba Extract + 50% Gum Tragacanth

T3: 25% Aquafaba Extract + 100% Gum Tragacanth

Means sharing different letter are significantly different (p < 0.05)

Table S19. Analysis of Variance for Creaming Index

Source of Variation	SS	df	MS	F	P-value	F crit
**Treatment**	1.675912	3	0.558637	124.4047	2.56E-20	2.838745
**Storage Interval**	3.559483	4	0.889871	198.1681	8.58E-26	2.605975
**T*S**	0.856685	12	0.07139	15.89815	1.29E-11	2.003459
**Within**	0.179619	40	0.00449			
**Total**	6.271699	59				

*p<0.05; **p<0.01; ***p<0.001; ns: non-significant

Table S20. Fisher LSD for Creaming Index

Storage Days	Treatments				Mean±SE
	**T_0**	**T_1**	**T_2**	**T_3**	
**0**	0±0 l	0±0 l	0±0 l	0±0 l	**0±0 e**
**7**	0.31±0.01 gh	0.16±0.01 ijk	0.12±0.03 jk	0.06±0.01 kl	**0.16±0.1 d**
**14**	0.63±0.03 c	0.4±0.1 efg	0.23±0.03 hij	0.22±0.12 hij	**0.37±0.18 c**
**21**	0.92±0.02 b	0.45±0.02 de	0.43±0.13 def	0.24±0.1 hi	**0.51±0.27 b**
**28**	1.23±0.03 a	0.64±0.04 c	0.54±0.13 cd	0.33±0.13 fgh	**0.69±0.36 a**
**Mean±SE**	**0.62±0.45 a**	**0.33±0.24 b**	**0.26±0.22 c**	**0.17±0.15 d**	

T0: Control

T1: 15% Aquafaba Extract + 30% Gum Tragacanth

T2: 20% Aquafaba Extract + 50% Gum Tragacanth

T3: 25% Aquafaba Extract + 100% Gum Tragacanth

Means sharing different letter are significantly different ($p < 0.05$)

Table S21. Analysis of Variance for Physical Stability

Source of Variation	SS	df	MS	F	P-value	F crit
**Treatment**	105.8811	3	35.29371	20.05671	4.3E-08	2.838745

**Storage Interval**	435.8336	4	108.9584	61.91889	1.33E-16	2.605975
**T*S**	0.764082	12	0.063673	0.036184	1^{ns}	2.003459
**Within**	70.38782	40	1.759695			
**Total**	612.8666	59				

*p<0.05; **p<0.01; ***p<0.001; ns: non-significant

Table S22. Fisher LSD for Physical Stability

**Storage Days**	**Treatments**				**Mean±SE**
	**T_0**	**T_1**	**T_2**	**T_3**	
**0**	98.67±1.29 ^{ab}	96±1.29 ^{cde}	98.01±1.29 ^{abc}	100.12±1.29 ^{a}	**98.2±1.9 ^{a}**
**7**	96.72±1.29 ^{bcd}	94.18±1.29 ^{efg}	96.16±1.29 ^{cde}	98.1±1.29A ^{bc}	**96.29±1.84 ^{b}**
**14**	94.77±1.29 ^{def}	92.36±1.29 ^{ghij}	94.3±1.29 ^{efg}	96.08±1.29 ^{cde}	**94.38±1.78 ^{c}**
**21**	92.82±1.29 ^{fghi}	90.54±1.29 ^{jk}	92.45±1.29 ^{ghij}	94.06±1.29 ^{efgh}	**92.47±1.72 ^{d}**
**28**	90.87±1.29 ^{ij}	88.72±1.29 ^{k}	90.59±1.29 ^{jk}	92.03±1.29 ^{hij}	**90.56±1.66 ^{e}**
**Mean±SE**	**94.77±3.06 ^{b}**	**92.36±2.88 ^{c}**	**94.3±2.93 ^{b}**	**96.08±3.16 ^{a}**	

T0: Control

T1: 15% Aquafaba Extract + 30% Gum Tragacanth

T2: 20% Aquafaba Extract + 50% Gum Tragacanth

T3: 25% Aquafaba Extract + 100% Gum Tragacanth

Means sharing different letter are significantly different ($p < 0.05$)

Table S23. Analysis of Variance for Heat Stability

**Source of Variation**	**SS**	**df**	**MS**	**F**	**P-value**	**F crit**
**Treatment**	84.03295	3	28.01098	16.42037	4.15E-07	2.838745

**Storage Interval**	249.7031	4	62.42577	36.59472	7.18E-13	2.605975
**T*S**	326.4508	12	27.20423	15.94744	1.23E-11	2.003459
**Within**	68.23473	40	1.705868			
**Total**	728.4215	59				

*p<0.05; **p<0.01; ***p<0.001; ns: non-significant

Table S24. Fisher LSD for Heat Stability

**Storage Days**	**Treatments**				**Mean±SE**
	**T_0**	**T_1**	**T_2**	**T_3**	
**0**	98.55±1.29 [a]	96.1±1.29 [cde]	97.88±1.29 [bcd]	100.33±1.29 [a]	**98.22±1.93 [a]**
**7**	95.91±1.29 [de]	94.45±1.29 [ef]	96.16±1.29 [cde]	98.15±1.29 [bc]	**96.16±1.76 [b]**
**14**	93.26±1.29 [fg]	92.79±1.29 [fgh]	94.43±1.29 [ef]	95.96±1.29 [de]	**94.11±1.69 [c]**
**21**	90.61±1.29 [ij]	91.13±1.29 [ghij]	92.71±1.29 [fghi]	93.77±1.29 [f]	**92.06±1.71 [d]**
**28**	87.96±1.29 [k]	89.47±1.29 [jk]	90.98±1.29 [hij]	91.58±1.29 [ghij]	**90±1.84 [e]**
**Mean±SE**	**93.26±4.03 [c]**	**92.79±2.66 [c]**	**94.43±2.75 [b]**	**95.96±3.38 [a]**	

T0: Control

T1: 15% Aquafaba Extract + 30% Gum Tragacanth

T2: 20% Aquafaba Extract + 50% Gum Tragacanth

T3: 25% Aquafaba Extract + 100% Gum Tragacanth

Means sharing different letter are significantly different ($p < 0.05$)

Table S25. Analysis of Variance for Phosphomolybdate Assay

**Source of Variation**	**SS**	**df**	**MS**	**F**	**P-value**	**F crit**
**Treatment**	13583.04	3	4527.679	2706.916	3.29E-46	2.838745

**Storage Interval**	610.0684	4	152.5171	91.18382	1.5E-19	2.605975
**T*S**	264.6937	12	22.05781	13.18747	2.29E-10	2.003459
**Within**	66.90533	40	1.672633			
**Total**	14524.7	59				

*p<0.05; **p<0.01; ***p<0.001; ns: non-significant

Table S26. Fisher LSD for Phosphomolybdate Assay

**Storage Days**	**Treatments**				**Mean±SE**
	**T_0**	**T_1**	**T_2**	**T_3**	
**0**	9.23±1.29 [m]	19.7±1.29 [i]	39.17±1.29 [e]	52.02±1.29 [a]	**30.03±17.41 [a]**
**7**	9.09±1.29 [m]	17.3±1.29 [j]	34.83±1.29 [f]	49.87±1.29 [b]	**27.77±16.53 [b]**
**14**	8.94±1.29 [m]	14.91±1.29 [k]	30.5±1.29 [g]	47.71±1.29 [c]	**25.52±15.75 [c]**
**21**	8.8±1.29 [m]	12.52±1.29 [l]	26.16±1.29 [h]	45.59±1.29 [c]	**23.27±15.1 [d]**
**28**	8.65±1.29 [m]	10.12±1.29 [m]	21.82±1.29 [i]	43.45±1.29 [d]	**21.01±14.59 [e]**
**Mean±SE**	**8.94±1.11 [d]**	**14.91±3.67 [c]**	**30.5±6.44 [b]**	**47.73±3.32 [a]**	

T0: Control

T1: 15% Aquafaba Extract + 30% Gum Tragacanth

T2: 20% Aquafaba Extract + 50% Gum Tragacanth

T3: 25% Aquafaba Extract + 100% Gum Tragacanth

Means sharing different letter are significantly different ($p < 0.05$)

Table S27. Analysis of Variance for DPPH Assay

**Source of Variation**	**SS**	**df**	**MS**	**F**	**P-value**	**F crit**
**Treatment**	11995.39	3	3998.463	2390.52	3.89E-45	2.838745
**Storage Interval**	1108.87	4	277.2174	165.7371	2.52E-24	2.605975

**T*S**	588.7172	12	49.05977	29.33085	4.78E-16	2.003459
**Within**	66.90533	40	1.672633			
**Total**	13759.88	59				

*p<0.05; **p<0.01; ***p<0.001; ns: non-significant

Table S28. Fisher LSD for DPPH Assay

**Storage Days**	**Treatments**				**Mean±SE**
	**T_0**	**T_1**	**T_2**	**T_3**	
**0**	9.57±1.29 ^{no}	16.93±1.29 ^{ij}	34.01±1.29 ^{e}	57.01±1.29 ^{a}	**29.38±19.09 ^{a}**
**7**	9.1±1.29 ^{no}	15.3±1.29 ^{jk}	30.1±1.29 ^{f}	51.92±1.29 ^{b}	**26.61±17.26 ^{b}**
**14**	8.63±1.29 ^{no}	13.68±1.29 ^{kl}	26.2±1.29 ^{g}	46.85±1.29 ^{c}	**23.84±15.44 ^{c}**
**21**	8.16±1.29 ^{o}	12.06±1.29 ^{lm}	22.3±1.29 ^{h}	36.68±1.29 ^{d}	**19.8±11.57 ^{d}**
**28**	7.69±1.29 ^{o}	10.45±1.29 ^{mn}	18.4±1.29 ^{i}	34.01±1.29 ^{e}	**17.64±10.75 ^{e}**
**Mean±SE**	**8.63±1.29 ^{d}**	**13.69±2.61 ^{c}**	**26.21±5.82 ^{b}**	**45.3±9.15 ^{a}**	

T0: Control

T1: 15% Aquafaba Extract + 30% Gum Tragacanth

T2: 20% Aquafaba Extract + 50% Gum Tragacanth

T3: 25% Aquafaba Extract + 100% Gum Tragacanth

Means sharing different letter are significantly different ($p < 0.05$)

Table S29. Analysis of Variance for Mold Count

**Source of Variation**	**SS**	**df**	**MS**	**F**	**P-value**	**F crit**
**Treatment**	22.786	3	7.595333	3.547006	0.022808	2.838745
**Storage Interval**	1.102933	4	0.275733	0.128767	0.009711	2.605975
**T*S**	1.5088	12	0.125733	0.058717	0.01	2.003459

**Within**	85.65347	40	2.141337
**Total**	111.0512	59	

*p<0.05; **p<0.01; ***p<0.001; ns: non-significant

Table S30. Fisher LSD for Mold Count

Storage Days	Treatments				Mean±SE
	**T_0**	**T_1**	**T_2**	**T_3**	
**0**	4.14±1.29 [a]	3.04±1.29 [a]	2.74±1.29 [a]	2.44±1.29 [a]	**3.09±1.29 [a]**
**7**	4.24±1.29 [a]	3.14±1.29 [a]	2.84±1.29 [a]	2.54±1.29 [a]	**3.19±1.29 [a]**
**14**	4.34±1.29 [a]	3.24±1.29 [a]	2.94±1.29 [a]	2.64±1.29 [a]	**3.29±1.29 [a]**
**21**	4.44±1.29 [a]	3.34±1.29 [a]	3.04±1.29 [a]	2.74±1.29 [a]	**3.39±1.29 [a]**
**28**	4.44±1.29 [a]	2.64±1.29 [a]	3.14±1.29 [a]	2.84±1.29 [a]	**3.27±1.32 [a]**
**Mean±SE**	**4.32±1.1 [a]**	**3.08±1.12 [b]**	**2.94±1.1 [b]**	**2.64±1.1 [b]**	

T0: Control

T1: 15% Aquafaba Extract + 30% Gum Tragacanth

T2: 20% Aquafaba Extract + 50% Gum Tragacanth

T3: 25% Aquafaba Extract + 100% Gum Tragacanth

Means sharing different letter are significantly different ($p < 0.05$)

Table S31. Analysis of Variance for Viscosity

Source of Variation	SS	df	MS	F	P-value	F crit
**Treatment**	1986.559	3	662.1862	395.9714	9.16E-30	2.838745
**Storage Interval**	88.94139	4	22.23535	13.2962	5.6E-07	2.605975
**T*S**	22.5957	12	1.882975	1.125974	0.036738	2.003459
**Within**	66.89234	40	1.672308			
**Total**	2164.988	59				

*p<0.05; **p<0.01; ***p<0.001; ns: non-significant

Table S32. Fisher LSD for Viscosity

Storage Days	Treatments				Mean±SE
	T_0	T_1	T_2	T_3	
**0**	4.99±1.29 ^{e}	3.91±1.29 ^{efg}	5.06±1.29 ^{e}	19.81±1.29 ^{a}	**8.44±6.96 ^{a}**
**7**	4.54±1.29 ^{ef}	3.27±1.29 ^{efgh}	4.29±1.29 ^{ef}	18.23±1.29 ^{ab}	**7.58±6.53 ^{ab}**
**14**	4.09±1.29 ^{efg}	2.63±1.29 ^{fgh}	3.51±1.29 ^{efg}	16.64±1.29 ^{bc}	**6.72±6.11 ^{bc}**
**21**	3.65±1.29 ^{efg}	1.99±1.29 ^{gh}	2.74±1.29 ^{fgh}	15.06±1.29 ^{cd}	**5.86±5.69 ^{cd}**
**28**	3.2±1.29 ^{efgh}	1.35±1.29 ^{h}	1.97±1.29 ^{gh}	13.47±1.29 ^{d}	**5±5.27 ^{d}**
**Mean±SE**	**4.09±1.27 ^{b}**	**2.63±1.44 ^{c}**	**3.51±1.57 ^{bc}**	**16.64±2.57 ^{a}**	

T0: Control

T1: 15% Aquafaba Extract + 30% Gum Tragacanth

T2: 20% Aquafaba Extract + 50% Gum Tragacanth

T3: 25% Aquafaba Extract + 100% Gum Tragacanth

Means sharing different letter are significantly different ($p < 0.05$)

Table S33. Analysis of Variance for Firmness

Source of Variation	SS	df	MS	F	P-value	F crit
**Treatment**	11448.56	3	3816.187	2283.173	9.71E-45	2.838745
**Storage interval**	1915.942	4	478.9856	286.5706	7.34E-29	2.605975
**T*S**	215.9583	12	17.99652	10.76707	4.42E-09	2.003459
**Within**	66.85762	40	1.67144			
**Total**	13647.32	59				

*p<0.05; **p<0.01; ***p<0.001; ns: non-significant

Table S34. Fisher LSD for Firmness

Storage Days	Treatments				Mean±SE
	T_0	T_1	T_2	T_3	
**0**	111.58±1.29 ^{d}	89.88±1.29 ^{j}	98.9±1.29 ^{gh}	118.88±1.29 ^{a}	**104.81±11.75 ^{a}**
**7**	107.89±1.29 ^{e}	84.36±1.29 ^{k}	94.11±1.29 ^{i}	116.92±1.29 ^{ab}	**100.82±13.1 ^{b}**
**14**	104.19±1.29 ^{f}	78.83±1.29 ^{l}	89.32±1.29 ^{j}	114.95±1.29 ^{bc}	**96.82±14.47 ^{c}**
**21**	100.49±1.29 ^{g}	73.3±1.29 ^{m}	84.53±1.29 ^{k}	112.99±1.29 ^{cd}	**92.83±15.84 ^{d}**
**28**	96.79±1.29 ^{h}	67.77±1.29 ^{n}	79.73±1.29 ^{l}	111.02±1.29 ^{d}	**88.83±17.21 ^{e}**
**Mean±SE**	**104.19±5.52 ^{b}**	**78.83±8.16 ^{d}**	**89.32±7.1 ^{c}**	**114.95±3.08 ^{a}**	

T0: Control

T1: 15% Aquafaba Extract + 30% Gum Tragacanth

T2: 20% Aquafaba Extract + 50% Gum Tragacanth

T3: 25% Aquafaba Extract + 100% Gum Tragacanth

Means sharing different letter are significantly different ($p < 0.05$)

Table S35. Analysis of Variance for Color

Source of Variation	SS	df	MS	F	P-value	F crit
**Treatment**	12.48202	3	4.160674	2.957728	0.043764	2.838745
**Storage interval**	1.305317	4	0.326329	0.23198	0.918764	2.605975
**T*S**	1.341433	12	0.111786	0.079466	0.999981	2.003459
**Within**	56.26852	40	1.406713			
**Total**	71.39729	59				

*p<0.05; **p<0.01; ***p<0.001; ns: non-significant

Table S36. Fisher LSD for Color

Storage Days	Treatments				Mean±SE
	T_0	T_1	T_2	T_3	
**0**	8.55±1.01 [a]	8.7±1.07 [a]	8.34±1.42 [abc]	8.46±1.24 [ab]	**8.51±1.03 [a]**
**7**	7.5±1.29 [abcd]	7.65±1.29 [abcd]	7.59±0.81 [abcd]	7.55±1.34 [abcd]	**7.57±1.03 [b]**
**14**	6.63±1.29 [bcde]	6.44±1.29 [de]	6.57±0.79 [cde]	6.7±1.03 [bcde]	**6.58±0.96 [c]**
**21**	5.36±1.29 [ef]	5.57±0.81 [ef]	5.44±0.88 [ef]	5.54±1.26 [ef]	**5.48±0.93 [d]**
**28**	4.57±0.81 [f]	4.37±0.81 [f]	4.45±1.12 [f]	5.46±1.02 [ef]	**4.71±0.93 [d]**
**Mean±SE**	**6.52±1.77 [a]**	**6.55±1.82 [a]**	**6.48±1.7 [a]**	**6.74±1.56 [a]**	

T0: Control

T1: 15% Aquafaba Extract + 30% Gum Tragacanth

T2: 20% Aquafaba Extract + 50% Gum Tragacanth

T3: 25% Aquafaba Extract + 100% Gum Tragacanth

Means sharing different letter are significantly different ($p < 0.05$)

Table S37. Analysis of Variance for Taste

Source of Variation	SS	df	MS	F	P-value	F crit
**Treatment**	12.54679	3	4.182263	3.041719	0.039848	2.838745
**Storage Interval**	0.35681	4	0.089202	0.064876	0.991957	2.605975
**T*S**	1.391177	12	0.115931	0.084316	0.999973	2.003459
**Within**	54.99867	40	1.374967			
**Total**	69.29344	59				

*p<0.05; **p<0.01; ***p<0.001; ns: non-significant

Table S38. Fisher LSD for Taste

Storage Days	Treatments	Mean±SE

	T_0	T_1	T_2	T_3	
**0**	8.55±1.2 [a]	8.45±0.88 [a]	8.33±0.81 [ab]	8.58±1.29 [a]	**8.48±0.92 [a]**
**7**	7.38±1.29 [abcd]	7.59±1.29 [abc]	7.45±0.88 [abc]	7.38±1.29 [abcd]	**7.45±1.03 [b]**
**14**	6.48±1.29 [cde]	6.42±1.29 [cde]	6.58±1.29 [bcde]	6.55±0.9 [bcde]	**6.51±1.03 [c]**
**21**	5.62±0.81 [def]	5.57±0.81 [ef]	5.51±0.81 [ef]	5.51±1.2 [ef]	**5.55±0.79 [d]**
**28**	4.37±1.29 [f]	4.39±0.81 [f]	4.37±1.29 [f]	4.52±0.81 [f]	**4.69±0.97 [d]**
**Mean±SE**	**6.52±1.74 [a]**	**6.48±1.73 [a]**	**6.45±1.69 [a]**	**6.69±1.54 [a]**	

T0: Control

T1: 15% Aquafaba Extract + 30% Gum Tragacanth

T2: 20% Aquafaba Extract + 50% Gum Tragacanth

T3: 25% Aquafaba Extract + 100% Gum Tragacanth

Means sharing different letter are significantly different ($p < 0.05$)

Table S39. Analysis of Variance for Odor

Source of Variation	SS	df	MS	F	P-value	F crit
**Treatment**	7.491541	3	2.49718	1.693907	0.01837	2.838745
**Storage Interval**	3.891575	4	0.972894	0.659941	0.032344	2.605975
**T*S**	5.315665	12	0.442972	0.30048	0.058568	2.003459
**Within**	58.96853	40	1.474213			
**Total**	75.66731	59				

*p<0.05; **p<0.01; ***p<0.001; ns: non-significant

Table S40. Fisher LSD for Odor

Storage Days	Treatments				Mean±SE
	T_0	T_1	T_2	T_3	

**0**	8.61±0.84 a	8.62±0.81 a	8.43±0.81 a	8.7±1.29 a	**8.59±0.83 a**
**7**	7.5±0.81 ab	7.34±0.81 abc	7.55±0.88 ab	7.41±1.04 ab	**7.45±0.76 b**
**14**	6.43±1.29 bcd	6.73±1.29 bcd	6.59±1.08 bcd	6.46±1.08 bcd	**6.56±1.02 c**
**21**	5.34±0.79 de	5.45±1.29 de	5.6±0.81 de	5.71±0.94 cde	**5.53±0.85 d**
**28**	4.63±1.29 e	4.52±0.81 e	4.4±1.05 e	5.53±0.8 de	**4.77±0.98 d**
**Mean±SE**	**6.52±1.79 a**	**6.53±1.72 a**	**6.51±1.67 a**	**6.74±1.43 a**	

T0: Control

T1: 15% Aquafaba Extract + 30% Gum Tragacanth

T2: 20% Aquafaba Extract + 50% Gum Tragacanth

T3: 25% Aquafaba Extract + 100% Gum Tragacanth

Means sharing different letter are significantly different ($p < 0.05$)

Table S41. Analysis of Variance for Texture

Source of Variation	SS	df	MS	F	P-value	F crit
**Treatment**	12.04214	3	4.014047	2.944371	0.044422	2.838745
**Storage Interval**	1.05419	4	0.263547	0.193317	0.019405	2.605975
**T*S**	1.322543	12	0.110212	0.080842	0.049979	2.003459
**Within**	54.5318	40	1.363295			
**Total**	68.95067	59				

*p<0.05; **p<0.01; ***p<0.001; ns: non-significant

Table S42. Fisher LSD for Texture

Storage Days	Treatments				Mean±SE
	**T_0**	**T_1**	**T_2**	**T_3**	
**0**	8.6±1.04 a	8.26±0.81 ab	8.35±0.81 a	8.73±0.81 a	**8.49±0.77 a**

**7**	7.43±1.29 abc	7.7±1.29 abc	7.75±1.23 abc	7.33±1.14 abc	**7.55±1.07 b**
**14**	6.41±0.81 cd	6.49±1.29 cd	6.58±1.29 bcd	6.52±0.68 cd	**6.5±0.9 c**
**21**	5.53±0.82 de	5.62±0.81 de	5.5±0.81 de	5.52±1.05 de	**5.54±0.75 d**
**28**	4.44±1.15 e	4.37±1.29 e	4.41±0.81 e	5.42±0.9 de	**4.66±1.01 e**
**Mean±SE**	**6.51±1.76 a**	**6.5±1.69 a**	**6.51±1.77 a**	**6.68±1.48 a**	

T0: Control

T1: 15% Aquafaba Extract + 30% Gum Tragacanth

T2: 20% Aquafaba Extract + 50% Gum Tragacanth

T3: 25% Aquafaba Extract + 100% Gum Tragacanth

Means sharing different letter are significantly different ($p < 0.05$)

Table S43. Analysis of Variance for Overall Acceptability

Source of Variation	SS	df	MS	F	P-value	F crit
**Treatment**	10.74881	3	3.582938	2.608106	0.054023	2.838745
**Storage Interval**	0.41884	4	0.10471	0.076221	0.009891	2.605975
**T*S**	1.148787	12	0.095732	0.069686	0.01	2.003459
**Within**	54.9508	40	1.37377			
**Total**	67.26724	59				

*p<0.05; **p<0.01; ***p<0.001; ns: non-significant

Table S44. Fisher LSD for Overall Acceptability

Storage Days	Treatments				Mean±SE
	**T_0**	**T_1**	**T_2**	**T_3**	
**0**	8.49±1.06 ^{a}	8.44±0.81 ^{a}	8.4±0.81 ^{a}	8.64±0.76 ^{a}	**8.64±0.75 ^{a}**
**7**	7.66±1.29 ^{ab}	7.71±1.29 ^{ab}	7.5±0.88 ^{ab}	7.62±0.95 ^{ab}	**7.62±0.96 ^{b}**
**14**	6.48±1.21 ^{bc}	6.54±1.29 ^{bc}	6.6±1.29 ^{bc}	6.37±1.01 ^{bc}	**6.5±1.03 ^{c}**
**21**	5.41±1.29 ^{cd}	5.04±0.88 ^{cd}	5.68±1.14 ^{cd}	5.53±0.68 ^{cd}	**5.42±0.91 ^{d}**
**28**	4.5±0.81 ^{d}	4.4±1.33 ^{d}	4.55±0.81 ^{d}	5.19±0.63 ^{cd}	**4.66±0.86 ^{d}**
**Mean±SE**	**6.54±1.82 ^{a}**	**6.46±1.78 ^{a}**	**6.52±1.72 ^{a}**	**6.64±1.47 ^{a}**	

T0: Control

T1: 15% Aquafaba Extract + 30% Gum Tragacanth

T2: 20% Aquafaba Extract + 50% Gum Tragacanth

T3: 25% Aquafaba Extract + 100% Gum Tragacanth

Means sharing different letter are significantly different ($p < 0.05$)
